# Exosomal miR-4645-5p from hypoxic bone marrow mesenchymal stem cells facilitates diabetic wound healing by restoring keratinocyte autophagy

**DOI:** 10.1093/burnst/tkad058

**Published:** 2024-01-17

**Authors:** Yan Shi, Shang Wang, Dewu Liu, Zhengguang Wang, Yihan Zhu, Jun Li, Kui Xu, Furong Li, Huicai Wen, Ronghua Yang

**Affiliations:** Department of Plastic, Medical Center of Burn Plastic and Wound Repair, The First Affiliated Hospital, Jiangxi Medical College, Nanchang University, Yongwaizheng Road, Donghu District, Nanchang, Jiangxi, 330006, China; Chongqing Key Laboratory of Traditional Chinese Medicine for Prevention and Cure of Metabolic Diseases, College of Traditional Chinese Medicine, Chongqing Medical University, Medical College Road, Yuzhong District, Chongqing, 400016, China; Medical Center of Burn Plastic and Wound Repair, The First Affiliated Hospital, Jiangxi Medical College, Nanchang University, Yongwaizheng Road, Donghu District, Nanchang, Jiangxi, 330006, China; Department of Orthopaedics, Peking University Third Hospital, 49 North Garden Road, Haidian District, Beijing, 100191 China; Department of Plastic and Aesthetic Surgery, Jiangxi Maternal and Child Health Hospital, Bayidadao Road, Donghu District, Nanchang 330006, China; HaploX Biotechnology Co., Ltd., Songpingshan Road, Nanshan District, Shenzhen 518057, Guangdong China; Key Laboratory of Xin’an Medicine, Ministry of Education, Anhui University of Chinese Medicine,Qianjiang Road, Yaohai District, Hefei 230038, Anhui, P. R. China; Translational Medicine Collaborative Innovation Center, Shenzhen People’s Hospital (The Second Clinical Medical College, Jinan University; The First Affifiliated Hospital, Southern University of Science and Technology), Dongmenbei Road, Luohu District, Shenzhen 518020, Guangdong, China; Department of Plastic, Medical Center of Burn Plastic and Wound Repair, The First Affiliated Hospital, Jiangxi Medical College, Nanchang University, Yongwaizheng Road, Donghu District, Nanchang, Jiangxi, 330006, China; Department of Burn and Plastic Surgery, Guangzhou First People's Hospital, South China University of Technology, Panfu Road, Yuexiu District, Guangzhou, Guangdong, 510180, China

**Keywords:** Bone marrow mesenchymal stem cell, AKT-mTORC1 signaling, Diabetic wound repair, Epidermal autophagy, Exosomes, MAPKAPK2, Migration, Proliferation, Re-epithelization, Keratinocyte

## Abstract

**Background:**

Refractory diabetic wounds are a common occurrence in patients with diabetes and epidermis-specific macroautophagy/autophagy impairment has been implicated in their pathogenesis. Therefore, identifying and developing treatment strategies capable of normalizing epidermis-specific macroautophagy/autophagy could facilitate diabetic wound healing. The study aims to investigate the potential of bone marrow mesenchymal stem cell-derived exosomes (BMSC-exos) from hypoxic conditions as a treatment to normalize epidermis-specific autophagy for diabetic wound healing.

**Methods:**

We compared the effects of bone marrow mesenchymal stem cell (BMSC)-sourced exosomes (BMSC-Exos) from hypoxic conditions to those of BMSC in normoxic conditions (noBMSC-Exos). Our studies involved morphometric assessment of the exosomes, identification of the microRNA (miRNA) responsible for the effects, evaluation of keratinocyte functions and examination of effects of the exosomes on several molecules involved in the autophagy pathway such as microtubule-associated protein 1 light chain 3 beta, beclin 1, sequestosome 1, autophagy-related 5 and autophagy-related 5. The experiments used human BMSCs from the American Type Culture Collection, an *in vivo* mouse model of diabetes (db/db) to assess wound healing, as well as the human keratinocyte HaCaT cell line. In the methodology, the authors utilized an array of approaches that included electron microscopy, small interfering RNA (siRNA) studies, RNA *in situ* hybridization, quantitative real-time reverse transcription PCR (qRT-PCR), the isolation, sequencing and differential expression of miRNAs, as well as the use of miR-4645-5p-specific knockdown with an inhibitor.

**Results:**

Hypoxia affected the release of exosomes from hypoxic BMSCs (hy-BMSCs) and influenced the size and morphology of the exosomes. Moreover, hyBMSC-Exo treatment markedly improved keratinocyte function, including keratinocyte autophagy, proliferation and migration. miRNA microarray and bioinformatics analysis showed that the target genes of the differentially expressed miRNAs were mainly enriched in ‘autophagy’ and ‘process utilizing autophagic mechanism’ in the ‘biological process’ category and miR-4645-5p as a major contributor to the pro-autophagy effect of hyBMSC-Exos. Moreover, mitogen-activated protein kinase-activated protein kinase 2 (MAPKAPK2) was identified as a potential target of exosomal miR-4645-5p; this was confirmed using a dual luciferase assay. Exosomal miR-4645-5p mediates the inactivation of the MAPKAPK2-induced AKT kinase group (comprising AKT1, AKT2, and AKT3), which in turn suppresses AKT-mTORC1 signaling, thereby facilitating miR-4645-5p-mediated autophagy.

**Conclusions:**

Overall, the results of this study showed that hyBMSC-Exo-mediated transfer of miR-4645-5p inactivated MAPKAPK2-induced AKT-mTORC1 signaling in keratinocytes, which activated keratinocyte autophagy, proliferation and migration, resulting in diabetic wound healing in mice. Collectively, the findings could aid in the development of a novel therapeutic strategy for diabetic wounds.

Highlights

- The study describes for the first time the paracrine effects and mechanisms of action of hyBMSC-Exos on keratinocytes and diabetic wounds.
- The study showed that hyBMSC-Exo-mediated transfer of miR-4645-5p inactivated MAPKAPK2-induced AKT-mTORC1 signaling in keratinocytes, which activated keratinocyte autophagy, proliferation and migration, resulting in diabetic wound healing in mice.
- The authors produced a comprehensive body of research with results that largely support their interpretation and their conclusions as related to the therapeutic potential of their findings.

## Background

Recently, there has been an increased risk of diabetic foot ulcers (DFUs) and dysfunctions in wound healing, with 1 in 4 diabetes patients at risk of both during their lifetime [1]. Both delayed and nonhealing DFUs can lead to amputation of the lower extremities and require additional prolonged treatment, resulting in increased medical costs and poor quality of life [2]. Therefore, studies are necessary to identify and develop effective agents capable of facilitating diabetic wound repair. Recent findings indicate that epidermal autophagy dysfunction is involved in the pathogenesis of impaired diabetic wound recovery [3,4]. Moreover, the role of epidermis-specific autophagy in the infiltration of immune cells and dermal granulation tissue formation and re-epithelization has been examined [5]. Given the importance of epidermal autophagy in cell functions and diabetic wound repair and regeneration, novel strategies capable of restoring epidermal autophagy may provide new opportunities for the treatment of diabetic wounds.

Owing to their self-renewal and multipotent differentiation abilities, bone marrow mesenchymal stem cells (BMSCs) have been recognized as an ideal source of adult stem-like cells and a viable option for treating various diseases, including immune system disease, myocardial infarction and osteochondral lesions [6–8]. BMSCs have been shown to promote tissue regeneration during wound healing by secreting chemical factors and directly differentiating into injured tissue [9]. Several findings indicate that BMSCs are preferred candidates for refractory diabetic wound treatment [10,11]. However, BMSC-based cell therapy is associated with some challenges, including the extremely low survival rate of directly transplanted stem cells in target tissues [12,13], malignant tumor formation and immune-mediated rejection [14,15]. Nevertheless, recent studies have shown that the paracrine activity of BMSCs might be associated with their role in the treatment of several diseases and that BMSC-sourced exosomes (BMSC-Exos) may play an essential role in this process [16].

Exosomes, which are derived from the invagination of endosomal membranes, are nanosized (30–200 nm) cell-sourced extracellular vesicles [17–19]. Previous studies have shown that exosomes exert therapeutic effects similar to or superior to those of directly transplanted stem cells and can overcome the limitations associated with direct transplantation of stem cells [20,21]. Exosomes exert biological effects through the transportation of specific proteins and RNAs from parent cells to target cells, and their content-loading selectivity is cell-specific and microenvironment-dependent [22]. Therefore, some strategies can be used to regulate and even augment the therapeutic potential of exosomes. Notably, culturing BMSCs in hypoxic environments has been shown to enhance the regenerative and cytoprotective effects of BMSCs [23], and the process is closely associated with the predominant paracrine activity of BMSCs in response to hypoxia [24]. The positive effects of hypoxic conditions on BMSCs could be attributed to the fact that *in vivo* BMSCs exist in the bone marrow with limited oxygen supply and hence have the capacity to adapt to hypoxic microenvironments. However, studies are yet to elucidate the effects and mechanisms of action of BMSC-Exos and hypoxic BMSC-sourced exosomes (hyBMSC-Exos) in keratinocytes and diabetic wound healing. Hence, here we examined the paracrine effects and mechanisms of action of hyBMSC-Exos on keratinocytes and diabetic wounds using molecular and transcriptomic techniques. The findings of this study could improve our understanding of the role of hyBMSC-Exos in wound healing and shed light on potential clinical applications.

## Methods

### Ethics statement

The study was approved by the committee of Shenzhen People’s Hospital and conducted in accordance with the NIH principles for the care and use of laboratory animals (NIH Pub. No. 85–23, revised 1996).

### BMSC culture and hypoxia treatment

Human BMSCs were obtained from American Type Culture Collection (ATCC, AC338194). The cells were cultured in a medium containing 125 pg/ml recombinant human fibroblast growth factor (rhFGF) basic, 15 ng/ml recombinant human insulin-like growth factor-1 (rhIGF-1), 7% fetal bovine serum (Gibco, A3160802) and 2.4 mM L-alanyl-L-glutamine using a mesenchymal stem cell growth kit for bone marrow-derived MSCs (ATCC, PCS-500-041). The cells were passaged into a new culture dish at 80% confluence. BMSCs after 5–6 passages were selected for further study. Before exosome isolation, the BMSCs were cultured in oxygen-controlled incubators (HuaYiNingChuang, Smartor 118 pro) under 5% CO_2_ and 74–94% N_2_ at 37°C for 48 h; the O_2_ concentration was maintained at 21 or 1% to simulate normoxic and hypoxic conditions, respectively.

### Exosome isolation and identification

The culture medium of treated BMSCs was centrifuged at 300 × *g* for 10 min, followed by centrifugation at 2000 × *g* for 15 min, and 12,000 × *g* for 30 min at 4°C to remove floating cells and cellular debris. Thereafter, the supernatant was passed through a 0.22-μm sterile filter (SteritopTM Millipore) and collected in 37.5 ml ultracentrifuge tubes (Beckman Coulter, Brea, CA, USA). The exosomes were concentrated by ultracentrifugation at 140,000 × *g* for 70 min at 4°C, using an SW32Ti rotor (Beckman Coulter, L-90 K with SW32Ti rotor). Subsequently, the ultra-filtered supernatant was washed twice with phosphate-buffered saline (PBS; Gibco, 20012–027) and re-centrifugated. Purified exosomes obtained after re-centrifugation at 140,000 × *g* for 70 min at 4°C were either stored at −80°C or used immediately in subsequent studies.

The size and morphology of exosomes obtained from hypoxia- and normoxia-treated BMSCs (hyBMSC-Exos and noBMSC-Exos, respectively) were determined using a Nanosight LM10 System (Nanosight Ltd, Navato, CA) and a transmission electron microscope (Philips, Tecnai 12). Exosomes were fixed in 2.5% glutaraldehyde for 30 min, placed on copper grids and then observed under the transmission electron microscope. A bicinchoninic acid (BCA) protein assay (Thermo Fisher Scientific, A53225) was performed to determine the protein concentration of the exosomes, which was quantified at 562 nm, using a microplate reader (Bio-Tek Instruments, ELx800).

### Generation of a mouse model of diabetic wound and exosome treatment

Male mice (8-week-old) with type-2 diabetes (db/db) were obtained from Shanghai Slac Laboratory Animal Co., Ltd (Shanghai, China) and maintained under 12-h light/dark conditions at 22 ± 2°C with free access to food and water for the duration of the experiment. After 1 week of acclimation, mice were randomly divided into five groups and anesthetized by isoflurane; full-thickness excisional wounds (8 mm) were made one on each side of the dorsal midline. Thereafter, the wounds were administrated with corresponding treatments at 0, 1 and 2 days post-wounding by subcutaneous injection into four mid-points of the wound edge: control group with PBS (100 μl), noBMSC-Exo group with noBMSC-Exos (10 μg in 100 μl of PBS), hyBMSC-Exo group with hyBMSC-Exos (10 μg in 100 μl of PBS), miR-NC/hyBMSC-Exo group with miR-NC/hyBMSC-Exos (10 μg in 100 μl of PBS) and miR-4645-5p^KD^/hyBMSC-Exo group with miR-4645-5p^KD^/hyBMSC-Exos (10 μg in 100 μl of PBS). All wounds were photographed at 0, 3, 6, 9 and 12 days post-wounding to observe the healing process, and the relative wound area was estimated using ImageJ software (NIH, Bethesda, MD, USA).

### Hematoxylin and eosin staining and immunohistochemical assay

The excised skin specimens were fixed in 4% paraformaldehyde solution for 24 h, embedded in paraffin and then subjected to hematoxylin and eosin (H&E) staining. Additionally, an immunohistochemical assay was performed to determine the expression of Ki67. Briefly, formalin-fixed paraffin-embedded skin biopsies were cut into 4-μm sections [25] and incubated with an antibody against Ki67 (Abcam, ab15580). The sections were stained with 3,3′-diaminobenzidine tetrahydrochloride (Yeasen, 36201ES03) and counterstained with H&E to detect the antibody reaction (brown reaction product). The stained sections were examined using a light microscope (Olympus, BX51, Japan).

### Western blotting analysis

Skin samples measuring 8 × 8 mm (wound and skin around wound) were collected from the mice and homogenized in RIPA lysis buffer (P0013, Beyotime). Cytosolic and nuclear fractions were extracted using a cytoplasmic and nuclear protein extraction kit (Beyotime Biotechnology, P0027), according to the manufacturer’s instructions. Protein quantification was performed using a BCA assay (P0012, Beyotime Biotechnology). Extracted proteins (50 μg) were then loaded and run on CFAS any KD PAGE gel (Zhonghuihecai, PE008, China) and then transferred onto PVDF membranes (Millipore, IPVH00010). The PVDF membranes were blocked at 22–25°C for 1 h and then incubated with primary antibodies overnight at 4°C. The primary antibodies included CD9 (Cell Signaling Technology, 98327), CD63 (Abcam, ab217345), CD81 (Cell Signaling Technology, 10037), tumor susceptibility 101 (TSG101; Abcam, ab125011), autophagy-related 5 (ATG5; HUABIO, ET1611–38), ATG7 (HUABIO, ET1610–53), MAP1LC3B (Abcam, ab192890), BECN1 (HUABIO, R1509–1), SQSTM1 (HUABIO, R1309–8), mitogen-activated protein kinase-activated protein kinase 2 (MAPKAPK2; Abcam, ab32567), PDAP1 (Abcam, ab204340), TAP2 (Abcam, ab180611), phospho-AKT (Thr308) (Cell Signaling Technology, 4056S), AKT (Cell Signaling Technology, 2920), phospho-ribosomal protein S6 kinase B1 (phospho-RPS6KB1) (Thr389) (Cell Signaling Technology, 9205), RPS6KB1 (Cell Signaling Technology, 9202), transcription factor EB (TFEB; Proteintech, 13372–1-AP), β-actin (Cell Signaling Technology, 3700 s), glyceraldehyde-3-phosphate dehydrogenase (GAPDH; 1:1000, CST, #5174S) and lamin B1 (1:1000, CST, #13435S). Thereafter, the membranes were incubated with secondary antibodies (Abcam, ab205718 and ab205719) for 1 h at 22–25°C, and the protein bands were visualized and detected using enhanced chemiluminescent detection substrate (WBKLS0500, Millipore, USA) and MultiImage Light Cabinet Filter Positions (Alpha Innotech, San Leandro, CA, USA), respectively. The intensity of each band was quantified using ImageJ software.

### Immunofluorescence staining

The tissue slides were incubated with primary anti-keratin 14 (anti-K14; Proteintech, 60320–1-Ig) and anti-ATG5 (HUABIO, ET1611–38) or anti-ATG7 (HUABIO, ET1610–53) at 4°C overnight for double immunofluorescence staining. Thereafter, the tissue slides were washed three times using PBS, followed by incubation with fluorescent secondary antibodies (Zhonghuihecai, PB004, PB005, PB006, PB007, China) for 1 h at 37°C. Nuclei were then stained with 4′,6-diamidino-2-phenylindole (DAPI; Sigma-Aldrich, D9542) and examined using a confocal microscope (Leica Microsystems, TCS SP8, Germany).

### Keratinocyte culture and treatment

The human keratinocyte (HaCaT) cell line was obtained from the Cell Bank of the Chinese Academy of Sciences (Shanghai, China) originally from ATCC (Manassas). The cells were cultured in minimum essential medium (Procell Life Science & Technology, PM150410) supplemented with 10% fetal bovine serum (Gibco, A3160802), 1% penicillin and streptomycin (Gibco, 15070–063), and 25 mmol/l glucose. The cells (5 × 10^6^) were further treated with noBMSC-Exos (100 μg/ml), hyBMSC-Exos (100 μg/ml) or PBS (control) for 24 h.

### Exosome uptake by HaCaT cells

The rate of uptake of hyBMSC-Exos and noBMSC-Exos by HaCaT cells was determined using 1,1-dioctadecyl-3,3,3,3-tetramethyl indotricarbocyanine (DIR)-labeled exosomes, according to the manufacturer’s instructions. Briefly, 4 mg/ml of DIR solution (Bioscience, D4006) was added to PBS and incubated with exosomes. Excess dye was removed from the labeled exosomes by ultracentrifugation at 100,000 × *g* for 1 h at 4°C. After final washing with PBS, the DIR-labeled exosomes were co-cultured with HaCaT cells for 24 h as the fluorescence intensity of cells observed at 3, 6, 12 and 24 h was strongest at 24 h. Fluorescence images of the DIR-labeled cells were observed using a confocal microscope (Leica Microsystems, TCS SP8, Germany) and the exosome uptake rate of the cells was assessed by measuring the fluorescence intensity of DIR.

### Proliferation assay

The proliferation rate of the keratinocytes after treatment with the exosomes for 24 h was determined using Cell Titer 96® Aqueous One Solution Cell Proliferation Assay [3-(4,5-dimethylthiazol-2-yl)-5-(3-carboxymethoxyphenyl)-2-(4-sulfophenyl)-2H-tetrazolium (MTS)] Promega (Madison, WI, USA) and 5-ethynyl-2′-deoxyuridine (EdU) assay (Beyotime Biotechnology, China) kits, according to the manufacturers’ instructions. EdU-stained images were captured using a fluorescence microscope (Leica Microsystems, TCS SP8, Germany).

### Migration assay

A wound healing assay was performed to measure HaCaT cell migration. Briefly, HaCaT cells were plated on 6-well plates at a density of 2 × 10^5^ cells/well and then grown to 100% confluence. To inhibit proliferation, the plated cells were incubated with mitomycin-C (S8146, Selleck, final concentration 5 μg/ml) at 37°C for 2 h, and cell monolayers were wounded using a sterile 200 μl pipette tip as previously described [26]. Thereafter, exosomes or PBS were added to the medium, and images were monitored at 0 and 24 h after scratching using an inverted light microscope (Olympus, BX51, Japan). Finally, the cell migration ability was estimated based on the residual wound and relative migration rates. Residual wound rate (%) = (A/B) × 100 (A, residual scratch width; B, original scratch width). Migration rate (%) =(B-A)/B× 100 . Relative migration rate (%) = C/D × 100 (C migration rate of the experimental group; D, migration rate of the control group).

### Small interfering RNA/miRNA transfection

For RNA interference, 10^5^ HaCaT cells and BMSCs were inoculated on 6-well plates with a cell density of 60–70%. Subsequently, the cells were transfected with 10 nM small interfering RNA (siRNA) each for *ATG5*, *ATG7*, argonaute RNA-induced silencing complex (RISC) catalytic component 2 (*AGO2*) or their scrambled control (GenePharma, Shanghai, China), using Lipofectamine 2000 (Invitrogen, USA), according to the manufacturer’s instructions. micrOFF ™ miRNA inhibitor is a specially modified miRNA inhibitor synthesized chemically to inhibit the expression of miRNAs in cells. Exosomes from BMSCs were transfected with 50 nM of miR-4645-5p inhibitor to knock down miR-4645-5p, using Lipofectamine 2000 transfection reagent (Thermo Fisher Scientific, 11668019). An equal concentration of a nontargeting control sequence (50 nM) was added to experimental samples as controls for non-sequence-specific effects in miRNA experiments. The miR-4645-5p inhibitor and miR-NC were purchased from Guangzhou RiboBio Co., Ltd. The sequence was as follows: inhibitor 5′-ACAAUAUUUCUUGCCUGGU-3′.

### Quantitative real-time PCR

Total RNA was extracted from cells and exosomes using AG RNAex Pro RNA reagent [Accurate Biotechnology (Hunan) Co., Ltd, AG21102] and reverse-transcribed to generate first-strand DNA, using miRNA first strand complementary DNA (cDNA) synthesis (tailing rReaction). Next, the synthesized cDNA was amplified using a real-time PCR system (StepOne Plus, ABI, USA) and specific primers (supplementary Table 1, see online supplementary material). Gene expression was normalized to *GAPDH* (internal control) and calculated using the 2^-ΔΔCT^ method [27].

### Exosomal miRNA microarray and bioinformatics analysis

The exosomal miRNA cargo sequencing for hyBMSC-Exos and noBMSC-Exos was performed by HaploX Biotech Company (Jiang Xi, China). Briefly, total RNA from Exos was extracted from the exosome pellets using an exosome RNA purification column kit (System Biosciences, USA), with three samples processed for each type of exosome. Libraries for small RNA sequencing were constructed before miRNA sequencing following the QIAseq miRNA library kit (cat#331505, Qiagen, Germany) standard protocol. The library was constructed in the GenCoding Lab (Guangzhou, China) and the miRNA expression profile was evaluated using an Illumina NGS system (MiSeq Personal Sequencer, NextSequence500, HiSeq 2500). The miRNA expression levels were normalized according to the expression of transcripts per million [28]. Differential expression analysis was performed with the DESeq2 package [29]. Candidate miRNAs between hyBMSC-Exos and noBMSC-Exos were assigned as differentially expressed miRNAs based on the following criteria: log_2_|fold change| > 1 and adjusted *p*-value <0.05. miRanda (http://www.microrna.org/microrna/home.do) and RNAhybrid (https://bibiserv.cebitec.uni-bielefeld.de/rnahybrid/) software [29] were used to predict the putative targets of differentially expressed 96 miRNAs. The ClusterProfiler package [30] was used to analyze the target genes of miRNAs described above for further Kyoto Encyclopedia of Genes and Genomes (KEGG) and gene ontology (GO) functional enrichment analysis.

### RNA fluorescence *in situ* hybridization

Following transcription, the RNA strands were labeled with 2-((1E,3E,5E)-5-(1-(5-CARBOXYPENTYL)-3,3-DIMETHYLINDOLIN-2-YLIDENE)PENTA-1,3-DIENYL)-1-ETHYL-3,3-DIMETHYL-3H-INDOLIUMCHLORIDE (Cy5) using the Label IT μArray Cy5 labeling kit (Mirus), with a labeling efficiency of 3 pmol of Cy5 dye per μg of miR-4646-5p, according to the manufacturer’s instructions (GE Healthcare). To prepare miR-4645-5p-lack exosomes, 100 μM miR-4645-5p-lack exosomes was transfected into BMSC cells, and 12 h later Cy5-labeled probes for miR-4645-5p junction and U6 were designed and synthesized by RiboBio Biotechnology (Guangzhou, China). Hybridization was performed in a humidified chamber at 37°C for 24 h after exosomes from BMSCs were added to the HaCaT cells. miR-4645-5p signals were detected using a fluorescent *in situ* hybridization kit (RiboBio Biotechnology, Guangzhou, China), while the nuclei were counterstained with DAPI, according to the manufacturer’s instructions. Fluorescence images were acquired using a confocal microscope (Leica Microsystems, TCS SP8, Germany).

### Luciferase reporter assay

Next, 5 × 10^4^ HaCaT cells were seeded into 24-well plates. At a cell convergence rate of 70%, the cells were transfected with firefly luciferase transcript containing either the wild-type (WT) or mutant form (MUT) of the 3’ untranslated regions (3’UTR) sequence of the mutated *MAPKAPK2* sequence in the presence of either miR-NC or miR-4645-5p^KD^. Subsequently, transient transfections were performed using Lipofectamine 2000 (Invitrogen, Karlspot, CA, USA). Luciferase activity was measured after 24 h using a dual luciferase reporter assay system (Promega, Madison, Wisconsin, USA) according to the manufacturer’s instructions and normalized to Renilla activity.

### Statistical analyses

The Shapiro–Wilk test was performed to test the normality of data. If the data met the normality, the independent samples t-test was used to determine if there was a significant difference between two sample means; otherwise, the Wilcoxon rank sum test was used. If data met the normality assumptions, one-way analysis of variance was conducted to test for significant differences among three or more groups; otherwise, the Kruskal–Wallis test was performed. If the differences between three or more groups were significant, the multiple independent samples t-test was used to test for significant differences between two groups, with Bonferroni correction. A two-sided *p* < 0.05 was considered statistically significant. All experiments were performed at least three times. All statistical analyses were performed using GraphPad Prism 8 software (GraphPad Software Inc., San Diego, CA, USA).

## Results

### Hypoxic preconditioning promotes BMSC-Exo secretion

**Figure 1:**
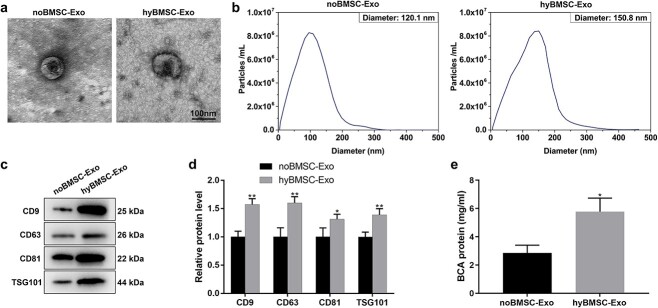
Hypoxia facilitates exosome production and release from BMSCs. (**a**) Transmission electron microscopy (TEM) revealed the morphology of noBMSC-Exos and hyBMSC-Exos (scale bar: 100 nm). (**b**) The size distribution of noBMSC-Exos and hyBMSC-Exos was examined using nanoparticle tracking analysis (NTA). (**c** and **d**) Western blotting analysis for exosome surface marker proteins, including CD9, CD63, CD81 and TSG101. The independent samples t-test was used to test for significant difference between two sample means. (**e**) A BCA assay was used to determine exosome protein concentration in the two groups. The data are presented as mean ± SD (n = 3). *p < 0.05, **p < 0.01. *BMSCs* bone marrow mesenchymal stem cells, *noBMSC-Exo* normoxic BMSC-sourced exosome, *hyBMSC-Exo* hypoxic BMSC-sourced exosome, *TSG101* tumor susceptibility 101, *BCA* bicinchoninic acid

Since BMSCs are naturally present in the bone marrow under hypoxic conditions of 1–8% O_2_ [31], we examined the effect of normoxic (21% O_2_; control) and hypoxic (1% O_2_) conditions on the secretion of exosomes by BMSCs. BMSCs were exposed to hypoxic or normoxic conditions for 48 h, followed by exosome isolation. Additionally, the size and morphology of hyBMSC-Exos and noBMSC-Exos were examined using transmission electron microscopy and nanoparticle tracking analysis (NTA). Transmission electron microscopy imaging revealed similar round-like shapes with different diameters between the two groups (Figure 1a). NTA revealed a larger size distribution in the hyBMSC-Exo group (average 150.8 nm) than in the noBMSC-Exo group (average 120.1 nm) (Figure 1b), indicating that hypoxia pretreatment affected the size of the exosomes. Furthermore, western blotting was performed to identify the expression of exosome surface markers, including tumor susceptibility 101 (TSG101), CD9, CD63 and CD81. The hyBMSC-Exo group showed significantly higher expression levels of the markers compared with the noBMSC-Exo group (Figure 1c, d). Moreover, the hyBMSC group had significantly higher concentrations of exosomal protein compared with the noBMSC-Exo group (Figure 1e) under the same protein loading conditions (Figure S1, see online supplementary material). Hypoxia promoted exosome production and secretion from BMSCs compared with that in the control group.

### hyBMSC-Exo facilitates diabetic wound healing

**Figure 2:**
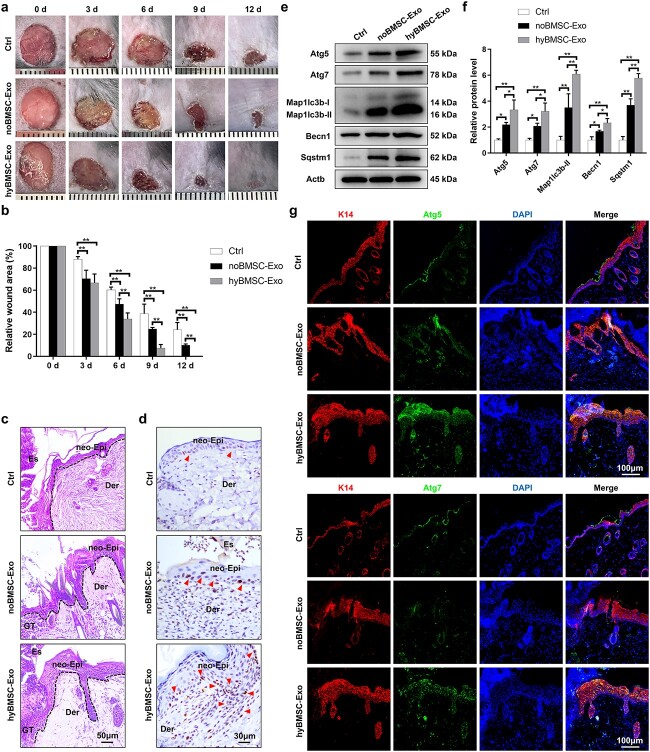
hyBMSC-Exo transplantation promotes diabetic wound healing. (**a**, **b**) Macroscopic view of wound healing and assessment of wound area in noBMSC-Exo-, hyBMSC-Exo- or PBS-treated mice (n = 6) at 0, 3, 6, 9 and 12 days post-wounding. (**c**) The wound healing rate was monitored in the three groups by H&E staining of skin sections at the wound edges at 6 days post-wounding (scale bar: 50 μm). The epithelium is marked with a dotted line. (**d**) Immunohistochemical analysis was used to determine Ki67 expression in wound sections in the three groups at 6 days post-wounding (scale bar: 30 μm). Arrowhesds indicate positive expression of Ki67. (**e**, **f**) Western blotting analysis for autophagy-associated proteins, including Atg5, Atg7, Map1lc3b-I/II, Becn1 and Sqstm1, in the three groups (n = 6). (**g**) Immunofluorescence staining for K14, Atg5 or Atg7 and DAPI staining of wound sections in the three groups at 6 days post-wounding (scale bar: 100 μm). The data are presented as mean ± SD. ^*^p < 0.05, ^**^p < 0.01. *BMSCs* bone marrow mesenchymal stem cells, *noBMSC-Exo* normoxic BMSC-sourced exosome, *hyBMSC-Exo* hypoxic BMSC-sourced exosome, *Ctrl* control, *Atg5* autophagy-related 5, *Map1lc3b-I/II* microtubule-associated protein 1 light chain 3 beta, *Becn1* beclin 1, *Sqstm1* sequestosome 1, *Actb* β-actin, *Der* dermis, *Es* eschar, *GT* granulation tissue, *neo-Epi* neo-epidermis, *K14* keratin 14

The therapeutic effect of hyBMSC-Exos in diabetic wounds was examined. Treatment with hyBMSC-Exos markedly accelerated the healing of full-thickness diabetic wounds compared with noBMSC-Exo and control treatments (Figure 2a, b, and Figure S2a, see online supplementary material). Additionally, H&E staining indicated a considerable increase in neo-epithelial tissue formation in the hyBMSC-Exo group when compared with the noBMSC-Exo and control groups at 6 days post-wounding (Figure 2c and Figure S2b). Moreover, the proliferation marker Ki67 was highly expressed in the regenerated epithelium in the hyBMSC-Exo group when compared with the noBMSC-Exo and control groups (Figure 2d and  Figure S2c), indicating that hyBMSC-Exos can increase keratinocyte proliferation. Furthermore, western blotting indicated that the hyBMSC-Exo group had significantly higher expression levels of autophagy-associated proteins, including Atg5, Atg7, microtubule-associated protein 1 light chain 3 beta (Map1lc3b-I/II), beclin 1 (Becn1) and sequestosome 1 (Sqstm1, the autophagy receptor) [4,32,33], when compared with the other two groups at 6 days post-wounding (Figure 2e, f). To further determine whether transplantation of the hyBMSC-Exos can influence keratinocyte-specific autophagy *in vivo*, skin samples from the mouse models were double-stained with autophagy markers (Atg5 and Atg7) and the keratinocyte marker keratin 14 (K14). The co-expression of K14 and Atg5 or Atg7 in the regenerated epidermis at 6 days post-wounding was significantly higher in the hyBMSC-Exo group than in the noBMSC-Exo and control groups (Figure 2g and  Figure S2d), indicating the superior pro-autophagy effect of the hyBMSC-Exos on keratinocytes.

### hyBMSC-Exo-induced autophagy is responsible for the activation of keratinocyte proliferation and migration

**Figure 3:**
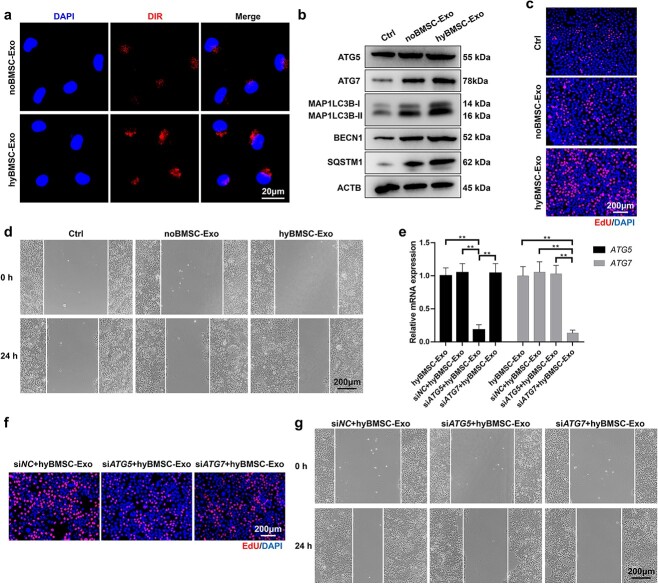
hyBMSC-Exo-induced autophagy contributes to the activation of HaCaT proliferation and migration. (**a**) Representative pictures of uptake of DIR-labeled noBMSC-Exos and hyBMSC-Exos by HaCaT cells (scale bar: 20 μm). (**b**) Western blotting analysis for autophagy-associated proteins. (**c**) HaCaT cell proliferation was assessed using EdU assay (scale bar: 200 μm). (**d**) HaCaT cell migration was measured using a wound healing assay (scale bar: 200 μm). (**e**) *ATG5* and *ATG7* mRNA levels in HaCaT cells stably transfected with si*NC*, si*ATG5* and si*ATG7* at 24 h following hyBMSC-Exo treatment was determined using qRT-PCR. (**f**) HaCaT cell proliferation 24 h after hyBMSC-Exo treatment was measured using EdU assay (scale bar: 200 μm). (**g**) HaCaT cell migration was assessed after hyBMSCs-Exo treatment using a wound-healing assay (scale bar: 200 μm). Data are presented as mean ± SD, all data are from n = 3 independent experiments. ^*^^*^p < 0.01. *BMSCs* bone marrow mesenchymal stem cells, *noBMSC-Exo* normoxic BMSC-sourced exosome, *hyBMSC-Exo* hypoxic BMSC-sourced exosome, *Ctrl* control, *DIR* 1,1-dioctadecyl-3,3,3,3-tetramethyl indotricarbocyanine, *ATG5* autophagy-related 5, *ATG7* autophagy-related 7, *MAP1LC3B-I/II* microtubule-associated protein 1 light chain 3 beta, *BECN1* beclin 1, *SQSTM1* sequestosome 1, *ACTB* β-actin, *EdU* 5-ethynyl-2′-deoxyuridine

In the present study, the effects of hyBMSC-Exos on keratinocyte proliferation and migration were examined. HaCaT cells were co-cultured with DIR-labeled noBMSC-Exos and hyBMSC-Exos in high glucose (HG) medium for 24 h and the uptake rate of the exosomes by HaCaT cells was determined. Representative images demonstrated that hyBMSC-Exo uptake by HaCaT cells was significantly higher than noBMSC-Exo uptake (Figure 3a, and Figure S3a, see online supplementary material**)**, indicating that hyBMSC-Exos were taken up more easily by HaCaT cells. Next, the expression of autophagy-associated proteins in HaCaT cells was examined after exposure to noBMSC-Exos or hyBMSC-Exos for 24 h. Exosome-treated HaCaT cells had significantly higher expression levels of the proteins when compared with untreated cells; however, hyBMSC-Exo-treated HaCaT cells showed the highest expression levels (Figure 3b and  Figure S3b**)**. To further confirm our findings in this study, HaCaT cells from three groups were double-stained with MAP1LC3B (green) and SQSTM1 (red). In line with the results of western blotting, the co-expression of MAP1LC3B and SQSTM1 in the HaCaT cells was higher in the hyBMSC-Exo group than in the noBMSC-Exo and control groups (Figure S3c**)**. Additionally, EdU assays and MTS showed that exosome treatments (noBMSC-Exos and hyBMSC-Exos) significantly promoted HaCaT proliferation compared with that in the control group; however, hyBMSC-Exo-treated HaCaT cells had the highest proliferation rate (Figure 3c and  Figure S3d). Furthermore, the wound healing and Matrigel (transwell) assays showed that the exosome treatments significantly enhanced the migration of HaCaT cells when compared with that in the untreated group; however, hyBMSC-Exo-treated HaCaT cells had the highest migration rates (Figure 3d and  Figure S3e–g). The present data indicated that the hyBMSC-Exos effectively potentiated keratinocyte functions, including autophagy, proliferation and migration.

HaCaT autophagy was inhibited by knocking down *ATG5* or *ATG7* to determine whether hyBMSC-Exo-induced HaCaT autophagy was responsible for the proliferation and migration of the cells. The successful knockdown of the genes was confirmed by qRT-PCR and western blotting (Figure 3e and  Figure S3h, i). Additionally, MTS, EdU, wound healing and Matrigel (transwell) assays showed that hyBMSC-Exo-induced proliferation and migration of HaCaT cells were largely suppressed by *ATG5* or *ATG7* knockdown (Figure 3f, g, and Figure S3j–m), indicating that HaCaT proliferation and migration were impaired along with the inhibition of cell autophagy. Overall, the results demonstrated the critical role of the pro-autophagy function of the hyBMSC-Exos in the activation of HaCaT proliferation and migration.

### Hypoxia treatment influences the miRNAs loaded into BMSC-Exos

**Figure 4:**
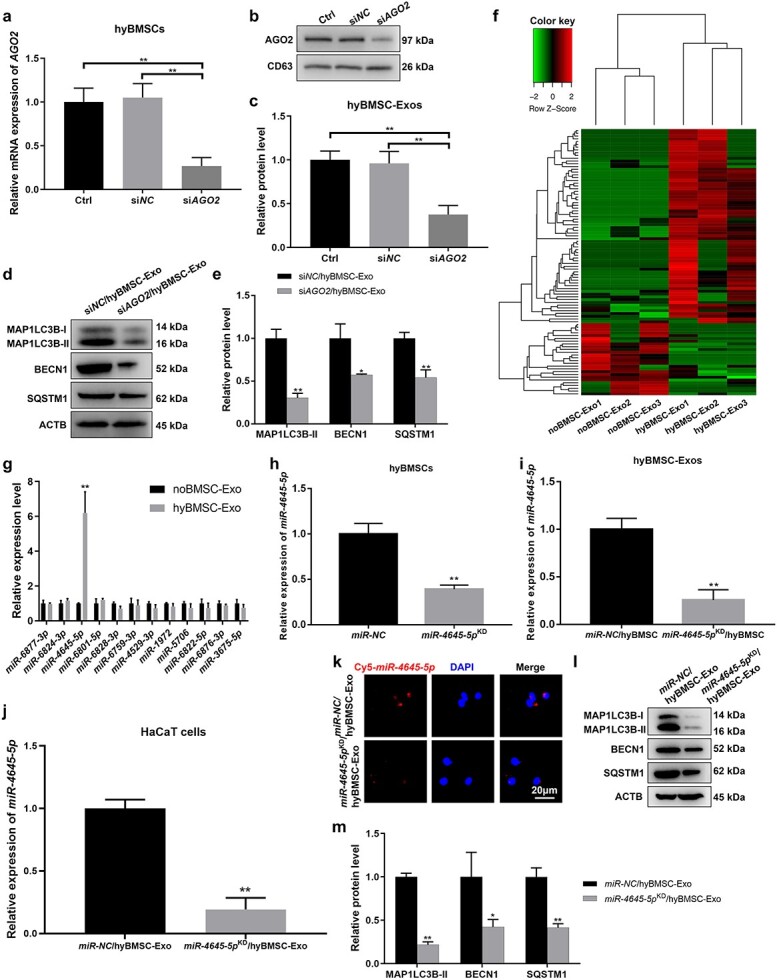
miR-4645-5p is a major contributor to the pro-autophagy effect of hyBMSC-Exos. (**a**) *AGO2* mRNA levels in BMSCs transfected with si*NC* or si*Ago2* and subjected to hypoxia conditions for 24 h was determined using qRT-PCR. (**b**, **c**) Western blotting analysis for AGO2 in exosomes from hyBMSCs transfected with si*NC* or si*AGO2*. (**d**, **e**) Western blotting analysis for MAP1LC3B-I/II, BECN1 and SQSTM1 in HaCaT cells after treatment with different hyBMSC-Exos for 24 h. (**f**) Heatmap of differentially expressed miRNAs depicting 70 upregulated and 26 downregulated miRNAs between noBMSC-Exo and hyBMSC-Exo groups. (**g**) mRNA expression levels of the top six upregulated and six downregulated miRNAs in noBMSC-Exos and hyBMSC-Exos were detected using qRT-PCR. (**h**) Confirmation of miR-4645-5p knockdown in hyBMSCs using qRT-PCR. (**i**) miR-4645-5p expression level in exosomes from hyBMSCs transfected with miR-NC (miR-NC/hyBMSC-Exo) or miR-4645-5p inhibitor (miR-4645-5p^KD^/hyBMSC-Exo). (**j**) The relative expression level of miR-4645-5p in target HaCaT cells after administration of miR-NC/hyBMSC-Exos or miR-4645-5p^KD^/hyBMSC-Exos. (**k**) Representative images of Cy5-labeled exosomal miR-4645-5p internalized by HaCaT cells after the administration of miR-NC/hyBMSC-Exos or miR-4645-5p^KD^/hyBMSC-Exos for 24 h (scale bar: 20 μm). (**l**, **m**) Western blotting analysis for MAP1LC3B-I/II, BECN1 and SQSTM1 in HaCaT cells after miR-NC/hyBMSC-Exo or miR-4645-5p^KD^/hyBMSC-Exo treatment for 24 h. Data are presented as mean ± SD, all data are from n = 3 independent experiments. ^*^p< 0.05, ^*^^*^p < 0.01. *BMSCs* bone marrow mesenchymal stem cells, *AGO2* argonaute 2, *miR/miRNA* microRNA, *hyBMSC-Exo* hypoxic BMSC-sourced exosome, Ctrl control, MAP1LC3B-I/II microtubule-associated protein 1 light chain 3 beta, *BECN1* beclin 1, *SQSTM1* sequestosome 1, *ACTB* β-actin

Exosomes exert biological effects by directly transferring specific proteins and RNAs to target cells [34]. AGO2, initially identified as a component of the RISC, plays important roles in the regulation of miRNA function by mediating the activity of miRNA-guided mRNA cleavage or via translational inhibition [35]. In the present study, silencing *AGO2* in hyBMSC-Exo suppressed the protein expression of the autophagy markers in HaCaT cells, confirming the essential role of exosomal miRNAs in keratinocyte autophagy (Figure 4a–e and  Figure S4a). miRNA sequencing of exosomes from hypoxia- and normoxia-treated BMSCs showed considerable differences in the composition of exosomal miRNAs, indicating that miRNAs were selectively packaged into exosomes after 48 h of hypoxia exposure (Figure 4f and  Figure S4b). Moreover, hypoxia can induce heterogeneous nuclear ribonucleoprotein and neutral sphingomyelinase 2 (nSMase2) enzyme expression [36], both of which can contribute to selectivity in exosomal miRNA loading [37,38]. GO analysis of target genes of the differentially expressed miRNAs indicated that they were significantly enriched in ‘autophagy’ and ‘process utilizing autophagic mechanism’ in the ‘biological process’ category (Figure S4c). Based on these results, we hypothesized that specific miRNAs from hyBMSC-Exos are responsible for the pro-autophagy effect of hyBMSC-Exos.

### Knockdown of miR-4645-5p affects the pro-autophagy effect of hyBMSC-Exos on HaCaT cells

The expression levels of the top six upregulated and downregulated miRNAs in the hyBMSC-Exo vs. noBMSC-Exo groups were examined by qRT-PCR and there was a significant increase in miR-4645-5p expression in the hyBMSC-Exo group when compared with that in the noBMSC-Exo group (Figure 4g). Successful knockdown of miR-4645-5p was confirmed by qRT-PCR (Figure 4h); moreover, there was a decrease in miR-4645-5p expression in exosomes from miR-4645-5p^KD^/hyBMSCs and miR-4645-5p^KD^/hyBMSC-Exo-treated HaCaT cells (Figure 4i, j). Consistent with the results of qRT-PCR, a Cy5-labeled immunofluorescence assay showed a marked decrease in miR-4645-5p immunofluorescence intensity in miR-4645-5p^KD^/hyBMSC-Exo-treated HaCaT cells when compared with that in the miR-NC/hyBMSC-Exo group (Figure 4k). Western blotting indicated that miR-4645-5p^KD^/hyBMSC-Exo treatment significantly decreased the protein levels of MAP1LC3B-I/II, BECN1 and SQSTM1 in HaCaT cells (Figure 4l, m). Combined with the results of cells co-expressing MAP1LC3B and SQSTM1 (Figure S5a, see online supplementary material), the current findings indicated the inhibition of HaCaT autophagy in the miR-4645-5p^KD^/hyBMSC-Exo group. Additionally, MTS, EdU, wound healing and Matrigel (transwell) assays indicated that knockdown of miR-4645-5p in hyBMSC-Exos suppresses HaCaT proliferation and migration (Figure S5b–f). Overall, the results indicated that miR-4645-5p was involved in the activation of HaCaT autophagy.

### Exosomal miR-4645-5p from hyBMSCs promotes HaCaT autophagy by inhibiting MAPKAPK2-induced AKT-mechanistic target of rapamycin kinase complex 1 signaling

**Figure 5:**
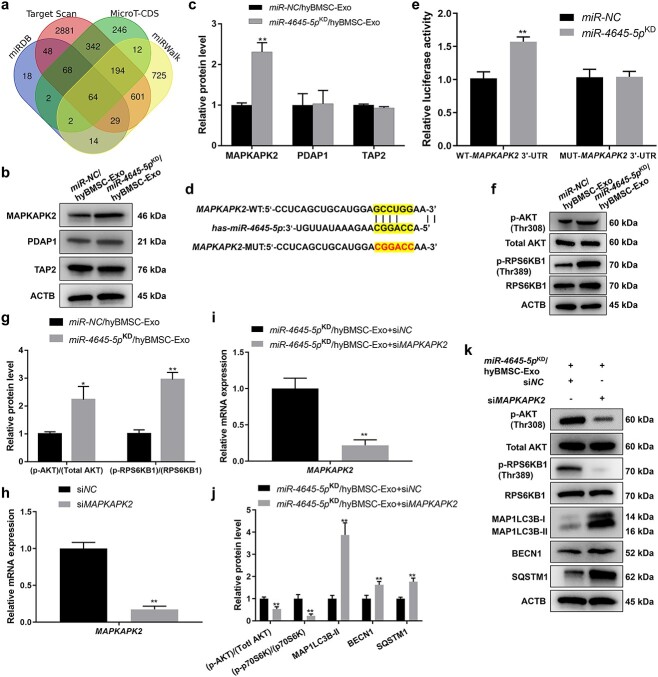
Exosomal miR-4645-5p promotes HaCaT autophagy by inhibiting MAPKAPK2-induced AKT-mTORC1 signaling. (**a**) Venn diagram showing miR-4645-5p target genes identified by four different independent microRNA-target-predicting programs (miRDB, TargetScan, MicroT-CDS and miRWalk). (**b**, **c**) Western blotting analysis of the expression levels of three predicted target genes in HaCaT cells after miR-NC/hyBMSC-Exos or miR-4645-5p^KD^/hyBMSC-Exo treatment for 24 h. (**d**) The predicted miR-4645-5p targeting sequence in the 3′-UTR of *MAPKAPK2*. (**e**) Luciferase reporter assay was performed to confirm that *MAPKAPK2* is the target gene of miR-4645-5p. (**f** and **g**) Western blotting analysis of the phosphorylation levels of AKT and RPS6KB1 in HaCaT cells after miR-NC/hyBMSC-Exos or miR-4645-5p^KD^/hyBMSC-Exo treatment for 24 h. (**h**) qRT-PCR for *MAPKAPK2* mRNA levels in HaCaT cells transfected with si*NC* or si*MAPKAPK2*. (**i**) qRT-PCR for *MAPKAPK2* mRNA levels in HaCaT cells transfected with si*NC* of si*MAPKAPK2* following 24 h of hyBMSC-Exo treatment. (**j** and **k**) Western blotting analysis of the phosphorylation levels of AKT and RPS6KB1 and autophagy-associated proteins in transfected HaCaT cells after miR-NC/hyBMSC-Exo or miR-4645-5p^KD^/hyBMSC-Exo treatment for 24 h. Data are presented as mean ± SD, all data are from n = 3 independent experiments. ^*^p < 0.05, ^*^^*^p < 0.01. *BMSCs* bone marrow mesenchymal stem cells, *miR/miRNA* microRNA, *MAPKAPK2* mitogen-activated protein kinase-activated protein kinase 2, *PDAP1* PDGFA-associated protein 1, TAP2 transporter 2, *RPS6KB1* ribosomal protein S6 kinase B1, *hyBMSC-Exo* hypoxic BMSC-sourced exosome, *MAP1LC3B-I/II* microtubule-associated protein 1 light chain 3 beta, *BECN1* beclin 1, *SQSTM1* sequestosome 1, *ACTB* β-actin, *AKT* AKT kinase group [AKT1 (AKT serine/threonine kinase 1), AKT2 and AKT3], mTORC1 mechanistic target of rapamycin kinase complex 1

To further elucidate the potential mechanism of action of exosomal miR-4645-5p in hyBMSC-Exo-induced HaCaT autophagy, four online databases, namely miRDB, TargetScan, miRWalk and MicroT-CDS, were independently searched to identify potential mRNA targets of miR-4645-5p. A total of 64 target genes were predicted by all four databases (Figure 5a). Thereafter, the expression levels of the 64 genes in miR-NC/hyBMSC-Exo- and miR-4645-5p^KD^/hyBMSC-Exo-treated HaCaT cells were determined by qRT-PCR. There was a significant increase in the expression of three genes, including *MAPKAPK2*, transporter 2 (*TAP2*) and PDGFA-associated protein 1 (*PDAP1*), in the miR-4645-5p^KD^/hyBMSC-Exo group (Figure S6). The previous study showed that miRNAs exert their biological effects on recipient cells by participating in a negative feedback loop to suppress signaling [39]. Western blotting showed that miR-4645-5p^KD^/hyBMSC-Exo treatment significantly increased the expression of MAPKAPK2 protein but did not significantly affect the protein expression levels of TAP2 and PDAP1 (Figure 5b, c). A 6-nucleotide long putative binding site of miR-4645-5p with the 3′UTR of MAPKPK2 was uncovered through analysis with the RNAhybrid2.0 site prediction tool (Figure 5d). Furthermore, a luciferase reporter assay was performed to determine whether *MAPKAPK2* 3′-UTR is a direct target of miR-4645-5p in HaCaT cells. Co-transfection with the *MAPKAPK2* WT luciferase construct decreased luciferase activity in the miR-4645-5p group, whereas co-transfection with the *MAPKAPK2*-MUT luciferase reporter did not significantly affect luciferase activity (Figure 5e).

Notably, MAPKAPK2 has been identified as an upstream positive regulator of AKT- mechanistic target of rapamycin kinase complex 1 (mTORC1) signaling [40]. KEGG pathway enrichment analysis showed the mTOR signaling pathway exhibited a significant change based on target genes of the differentially expressed miRNAs (Figure S4c). Given the known function of AKT-mTORC1 signaling as a negative regulator of autophagy [41], we speculated that exosomal miR-4645-5p from the hyBMSCs induced HaCaT autophagy through the inhibition of AKT-mTORC1 signaling. Bhaskar and Hay stated that it is necessary to phosphorylate AKT to achieve enzymatic activity and to induce mTORC1 activity, as estimated by the phosphorylation level of RPS6KB1 [42]. As expected, miR-4645-5p knockdown induced the activation of AKT-mTORC1 signaling, as evidenced by the phosphorylation levels of AKT and RPS6KB1 (Figure 5f, g). To further verify the link between exosomal miR-4645-5p and MAPKAPK2-induced AKT-mTORC1 signaling in the regulation of HaCaT autophagy, *MAPKAPK2* was silenced in HaCaT cells, which was confirmed by qRT-PCR (Figure 5h). Moreover, there was a decrease in *MAPKAPK2* expression in HaCaT cells treated with *miR-4645-5p*^KD^/hyBMSC-Exos (Figure 5i). Western blotting and immunofluorescent staining indicated that *MAPKAPK2* silencing decreased AKT and RPS6KB1 phosphorylation levels and increased the expression of autophagy markers in HaCaT cells treated with miR-4645-5p^KD^/hyBMSC-Exos (Figure 5j, k and  Figure S5g). Overall, the results indicated that exosomal miR-4645-5p induced HaCaT autophagy activity by suppressing AKT-mTORC1 signaling through *MAPKAPK2* inhibition.

### miR-4645-5p is involved in hyBMSC-Exo-induced diabetic wound healing

**Figure 6:**
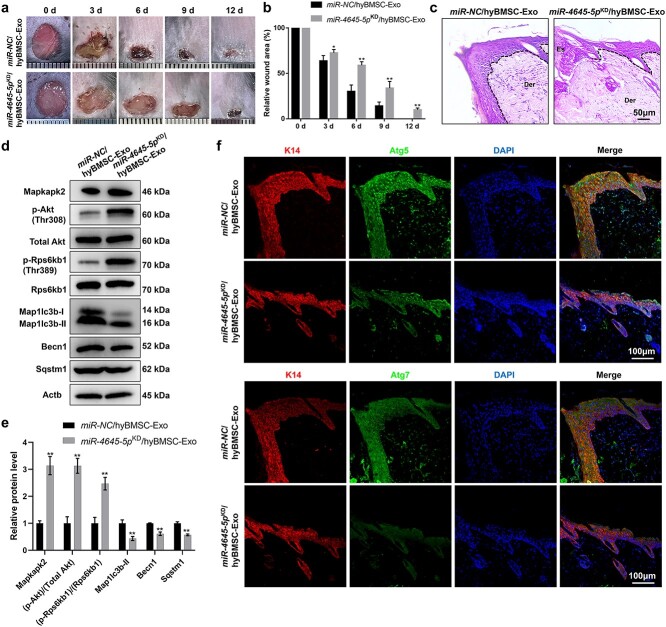
Exosomal miR-4645-5p contributes to the therapeutic effect of hyBMSC-Exos in diabetic wound healing. (**a**, **b**) Macroscopic view of wound healing and assessment of wound area in mice treated with miR-NC/hyBMSC-Exos or miR-4645-5p^KD^/hyBMSC-Exos (n = 6) at 0, 3, 6, 9 and 12 days post-wounding. (**c**) Wound healing rate was monitored in the three groups by H&E staining of skin sections at the wound edges at 6 days post-wounding (scale bar: 50 μm). The epithelium is marked with a dotted line. (**d**, **e**) Western blotting analysis of the phosphorylation levels of Akt and Rps6kb1 and autophagy-associated proteins in the two groups (n = 6). (**f**) Immunofluorescence staining for K14, Atg5 or Atg7, and DAPI staining of wound sections in the two groups at 6 days post-wounding (scale bar: 100 μm). Data are presented as mean ± SD. ^*^p < 0.05, ^*^^*^p < 0.01. *BMSCs* bone marrow mesenchymal stem cells, *Mapkapk2* mitogen-activated protein kinase-activated protein kinase 2, *Rps6kb1* ribosomal protein S6 kinase B1, *hyBMSC-Exo* hypoxic BMSC-sourced exosome, Atg5 autophagy-related 5, Map1lc3b-I/II microtubule-associated protein 1 light chain 3 beta, *Becn1* beclin 1, *Sqstm1* sequestosome 1, *AKT* AKT kinase group [AKT1 (AKT serine/threonine kinase 1) AKT2 and AKT3], *RPS6KB1* ribosomal protein S6 kinase B1, Actb β-actin, *Der* dermis, *Es* eschar, *neo-Epi* neo-epidermis, *K14* keratin 14

**Figure 7:**
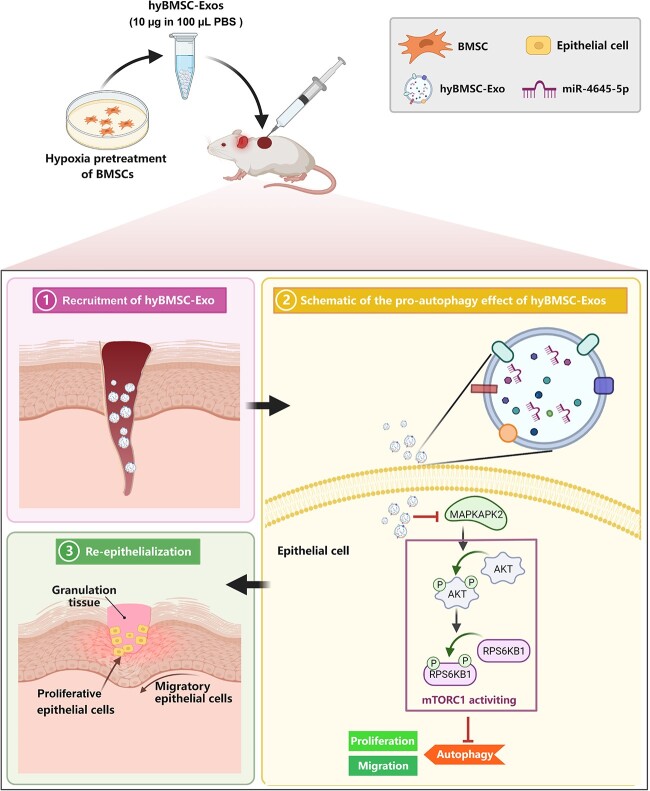
Schematic representation of the therapeutic effect of hyBMSC-Exos on diabetic wounds. The schematic graph shows that hyBMSC-Exos promotes wound healing by promoting keratinocyte autophagy. Specifically, hyBMSC-Exo-mediated transfer of miR-4645-5p inactivates MAPKAPK2-induced AKT-mTORC1 signaling in keratinocytes, leading to keratinocyte autophagy, proliferation and migration. *BMSC* bone marrow mesenchymal stem cell*, hyBMSC-Exo* hypoxic BMSC-sourced exosome, *AKT* AKT kinase group [AKT1 (AKT serine/threonine kinase 1), AKT2 and AKT3], *mTORC1* mechanistic target of rapamycin kinase complex 1, *RPS6KB1* ribosomal protein S6 kinase B1

To further verify the effect of exosomal miR-4645-5p on diabetic wound healing, we treated full-thickness wounds with exosomes from miR-4645-5p^KD^/hyBMSCs or miR-NC/hyBMSCs. There was a significant delay in wound healing in mice treated with miR-4645-5p^KD^/hyBMSCs-derived exosomes compared with that in mice treated with miR-NC/hyBMSC-Exos (Figure 6a, b, and Figure S7a, see online supplementary material). Additionally, H&E staining results indicated that re-epithelization was slower in mice treated with miR-4645-5p^KD^/hyBMSCs-derived exosomes than in mice treated with miR-NC/hyBMSC-Exos at 6 days post-wounding (Figure 6c and  Figure S7b). Furthermore, we assessed the status of Akt-mTORC1 signaling as well as the expression levels of MAPKAPK2 and autophagy-associated proteins in the two groups. MAPKAPK2 expression and Akt and RPS6KB1 phosphorylation increased with decreasing expression levels of autophagy-related protein in the miR-4645-5p^KD^/hyBMSC-Exo group when compared with those in the miR-NC/hyBMSC-Exo group (Figure 6d, e), thus indicating that treatment with miR-4645-5p^KD^/hyBMSC-Exo promoted the activation of MAPKAPK2-induced Akt-mTORC1 signaling, resulting in autophagy inhibition. Correspondingly, miR-4645-5p^KD^/hyBMSC-Exo treatment markedly inhibited the co-expression of Atg5 or Atg7 and K14 in the regenerated epidermis, indicating that epidermal autophagy was suppressed in the miR-4645-5p^KD^/hyBMSC-Exo group (Figure 6f and  Figure S7c). Overall, the results indicated that that hyBMSC-Exo-derived miR-4645-5p facilitated wound healing by regulating epidermal autophagy through the inhibition of MAPKAPK2-induced AKT-mTORC1 signaling (Figure 7).

## Discussion

DFUs are a major public health problem globally. Although BMSC-based cell therapy for diabetic wound regeneration has entered the clinical trial phase (http://clinicaltrials.gov), ethical regulations and the time and cost resources involved in the preparation of large quantities of stem cells have limited their clinical application. Compared with BMSC-based cell treatment, BMSC-derived exosome therapy has numerous advantages, including higher stability, lower manufacturing costs, and easier storage and infusion treatment [43]. According to the present study results, hypoxia affected both the structure and content of BMSC-Exo; moreover, hyBMSC-Exos regulated keratinocyte autophagy, proliferation and migration, thus promoting diabetic wound healing. Additionally, exosomal miR-4645-5p was identified as a key player in the pro-autophagy effect of hyBMSC-Exos, with MAPKAPK2-induced AKT-mTORC1 signaling acting negatively downstream of exosomal miR-4645-5p.

Previous studies have demonstrated that the structural and biochemical properties of exosomes are strongly affected by the microenvironment of the parent cells [44,45]. Although the effect of hypoxia on BMSCs has been studied [46], exosome biogenesis in BMSCs under hypoxic conditions is yet to be examined. In the present study, exosome surface protein markers were highly expressed in hyBMSC-Exos. Further analysis showed that hyBMSC-Exos were more easily captured by keratinocytes than noBMSC-Exos, which could be attributed to the higher expression of cell–exosome fusion proteins [47], including CD9 and CD81, on the surface of hyBMSC-Exos, when compared with those on noBMSC-Exos. Studies have shown that released exosomes are captured by target cells via ligand–receptor interactions, fusion with the plasma membrane or internalization into the endocytic compartment [48,49]. Moreover, hypoxia has been reported to directly impact cellular protein expression [50,51], which may directly or indirectly affect the specific surface protein composition of the exosomes.

Autophagy, a catabolic process essential for the degradation and recycling of misfolded proteins and damaged organelles, directly influences cell bioactivity and is related to disease progression [52]. A previous study revealed a higher expression of autophagy-related genes in the regenerated epidermis of wounded skin compared with that in normal skin [5]. At the cellular level, keratinocytes have been shown to be involved in skin repair and are responsible for epidermal regeneration through keratinocyte proliferation and migration [51,53]. Moreover, keratinocyte proliferation and migration are activated by epidermal autophagy, and dysfunction of keratinocyte autophagy impaired skin repair in rats [5]. Although epidermal autophagy dysfunction has been reported as an important pathophysiological feature of diabetic wounds, studies on potential treatments for improving epidermal autophagy in patients with diabetes are lacking. In the present study, hyBMSC-Exos facilitated keratinocyte functions, especially epidermal autophagy, thus promoting rapid diabetic wound closure. Additionally, silencing *ATG5* and *ATG7*, early markers of autophagy activation in keratinocytes, suppressed hyBMSC-Exo-induced keratinocyte proliferation and migration, indicating that the activation of keratinocyte proliferation and migration could be attributed to hyBMSC-Exo-induced autophagy. Overall, studies suggest that nutrients and energy [54] as well as DNA damage repair [55] from the autophagy process are partly involved in maintaining keratinocyte metabolism and homeostasis under extreme stress, such as HG conditions.

It is remarkable that SQSTM1 was employed to detect the status of autophagic flux in this study. However, the relationship between SQSTM1 andh autophagic flux remains controversial. Some studies reported that an increased level of SQSTM1 indicates blockage of autophagic flux [56]. In contrast to these studies, we observed significant inhibition of autophagy and downregulation of SQSTM1 in HG-treated HaCaT cells and db/db mouse wound models, which were both reversed by exosomes treatment. In line with our results, recent findings have revealed that HG led to a decrease in SQSTM1 and inhibition of autophagy in HaCaT cells, while knockdown of SQSTM1 reduced autophagic flux in HaCaT cells and delayed wound healing in mice [4]. Moreover, loss of SQSTM1 was found to contribute to inhibition of autophagosome formation and impairment of autophagic flux [57,58]. Considering that the role of SQSTM1 in autophagic flux is as an autophagy substrate, which binds to MAP1LC3B-II to induce autophagosome formation and is subsequently degraded after autophagosome formation, we hypothesized that variation in SQSTM1 expression level may be responsible for the relationship between SQSTM1 and autophagic flux. To further confirm this hypothesis, we monitored expression levels of MAP1LC3B-II and SQSTM1 in parallel in both HG-treated HaCaT cells and db/db mouse wound models with exosomes treatment or not, normal glucose (NG)-treated HaCaT cells or age-matched WT mouse wound models as a control. *In vivo* and *in vitro*, decreased levels of MAP1LC3B-II and SQSTM1 were observed in HG-treated HaCaT cells and db/db mouse wound models, compared with controls (Figure S8a–d, see online supplementary material). Notably, upregulation of MAP1LC3B-II and SQSTM1 expression levels by hyBMSC-Exos treatment in HG-treated HaCaT cells and db/db mouse wound models were not obviously different from that in HG-treated HaCaT cells and age-matched WT mouse wound models (Figure S8a–d). On the basis of the previous literature and our present study, deseases in SQSTM1 during HG concentrations attenuated autophagic flux, and hyBMSC-Exos treatment is suggested to promote autophagic flux by increasing SQSTM1. Thus, SQSTM1 is positively correlated with autophagic flux in this study.

Previous findings on the effects of mTORC1 on cell autophagy are inconsistent. The activation of mTORC1 during long-term starvation has been reported to be responsible for autophagic flux via the induction of autophagic lysosome reformation, thus maintaining lysosomal homeostasis [33,59,60]. In contrast, other studies have indicated that HG concentrations inhibit cell autophagy by activating mTORC1 [61,62], which is consistent with the findings of the present study. Overall, these findings indicate that the biological functions of mTORC1 in autophagy regulation are context dependent. Further, one potential explanation for the increased expression of the autophagy-related protein induced by mTORC1 inactivation [63,64] is that inhibition of mTORC1 is sufficient to increase nuclear translocation of TFEB and the transcription of TFEB-target genes, including *Atg5*, *Atg7*, *SQSTM1* and *BECN1*. Consistent with previous results, HaCaT cells transfected with MAPKAPK2 siRNA after miR-4645-5p^KD^/hyBMSC-Exo treatment had increased nuclear levels of TFEB (Figure S9a–d). Therefore, we speculated that the upregulation of autophagy-related protein expression by miR-4645-5p-mediated mTORC1 inhibition might be associated with the transcriptional activation of TFEB. The results further support our speculation, indicating that the expression levels of autophagy-associated proteins are markedly lowered in HaCaT cells transfected with *TFEB* siRNA subjected to treatment with the hyBMSC-Exos (Figure S9e–g). Furthermore, no expression of autophagy-related mRNA and other factors, including MAPKAPK2, AKT-mTOR1 signaling and TFEB, was detected in the hyBMSC-Exo and noBMSC-Exo (results not shown).

Wound healing is a multifaceted process involving immunomodulation, re-epithelization and resolution, which are orchestrated by various cells and their associated extracellular matrix. In addition to epidermal-specific autophagy, the biological effect of autophagy in other cell types has been reported during wound repair. For example, phases of repair also involve autophagy-initiated M1 macrophage survival and autophagy-triggered phenotypic shifts of fibroblasts [65,66]. Based on these findings, it is possible that hyBMSC-Exos induce autophagy in other cell types during wound healing; however, this warrants further study. Moreover, recent findings indicate that MAPKAPK2 can function as a positive modulator in inflammation, which has been demonstrated in psoriasis and lipopolysaccharide-induced liver damage [67,68]. Inhibition of MAPKAPK2 expression has been proposed as a therapeutic strategy for preventing excessive recruitment of monocytes/macrophages in damaged areas [69]. Considering the relationship between an excessive inflammatory response and refractory diabetic wounds [70], the results of the present study indicate that hyBMSC-Exo-based therapy targeting MAPKAPK2 activity may provide clinical strategies not only for keratinocyte autophagy restoration but also for the treatment of inflammatory disorders in diabetic wound healing. We were unable to elucidate the role of hyBMSC-Exos in wound-induced inflammation in the present study; this topic warrants further investigation. Moreover, only the top six upregulated and downregulated miRNAs were selected from a total of 96 differentially expressed miRNAs to study the therapeutic mechanism of action of hyBMSC-Exos in the present study, highlighting the need for future studies to examine the possible contributions of other miRNAs in hyBMSC-Exos.

## Conclusions

In summary, the results of this study showed that hyBMSC-Exo-mediated transfer of miR-4645-5p inactivated MAPKAPK2-induced AKT-mTORC1 signaling in keratinocytes, which promoted keratinocyte autophagy, proliferation and migration, resulting in diabetic wound healing. Overall, the findings could facilitate the development of a novel strategy for the treatment of DFUs.

## Abbreviations

ACTB/Actb: β-Actin; AGO2: Argonaute RNA-induced silencing complex catalytic component 2; AKT: AKT kinase group [AKT1 (AKT serine/threonine kinase 1), AKT2 and AKT3]; ATG/Atg: Autophagy-related; BECN1/Becn1: Beclin 1; BCA: Bicinchoninic acid; BMSCs: Bone marrow mesenchymal stem cells; BMSC-Exos: BMSC-sourced exosomes; cDNA: Complementary DNA; Cy5: 2-((1E,3E,5E)-5-(1-(5-CARBOXYPENTYL)-3,3-DIMETHYLINDOLIN-2-YLIDENE)PENTA-1,3-DIENYL)-1-ETHYL-3,3-DIMETHYL-3H-INDOLIUMCHLORIDE; DAPI: 4′,6-Diamidino-2-phenylindole; DFU: Diabetic foot ulcer; DIR: 1,1-Dioctadecyl-3,3,3,3-tetramethyl indotricarbocyanine; EdU: 5-Ethynyl-2′-deoxyuridine; GAPDH: Glyceraldehyde-3-phosphate dehydrogenase; GO: Gene ontology; HG: High glucose; H&E*:* Hematoxylin and eosin; K14: Keratin 14; KEGG: Kyoto Encyclopedia of Genes and Genomes; miRNA: MicroRNA; MAP1LC3B/Map1lc3b-I/II: Microtubule-associated protein 1 light chain 3 beta; MAPKAPK2/Mapkapk2: Mitogen-activated protein kinase-activated protein kinase 2; mTORC1: Mechanistic target of rapamycin kinase complex 1; MTS: 3-(4,5-Dimethylthiazol-2-yl)-5-(3-carboxymethoxyphenyl)-2-(4-sulfophenyl)-2H-tetrazolium; MUT: Mutant form; NTA: Nanoparticle tracking analysis; nSMase2: neutral sphingomyelinase 2; OD: Optical density; PBS: Phosphate-buffered saline; PDAP1: platelet derived growth factor subunit A-associated protein 1; RPS6KB1/Rps6kb1: Ribosomal protein S6 kinase B1; qRT-PCR: Quantitative real-time PCR; rhFGF: recombinant human fibroblast growth factor; rhIGF-1: recombinant human insulin-like growth factor-1; RISC: RNA-induced silencing complex; siNC: Negative control; siRNA: Small interfering RNA; SQSTM1/Sqstm1: Sequestosome 1; TAP2: Transporter 2; TFEB: Transcription factor EB; TSG101: Tumor susceptibility 101; 3’UTR: 3’ untranslated regions; WT: Wild type.

## Funding

This study was supported by the National Natural Science Foundation of China (No. 82060350, No. 82002272, No. 82272276), China Postdoctoral Science Foundation (No. 2022 M711335, No. 2021 M701434), GuangDong Basic and Applied Basic Research Foundation (No. 2022A1515110490, No. 2021A1515011453， No. 2022A1515011380, No. 2022A1515012160), Industry–university–research Innovation Fund of Higher Education of China (No. 2021JH028), the Science and Technology Innovation Committee of Shenzhen (No. JCYJ20220530152015036).

## Data availability

All data needed to support the conclusions are present in the paper and/or the supplementary materials. Additional data related to this paper may be requested from the authors.

## Authors’ contributions

FL, HW, RY contributed to conceptualization; YS, SW, DL, ZW, YZ, JL contributed to investigation; YS, SW, DL, KX contributed to formal analysis. All authors read and approved the final manuscript.

## Ethics approval and consent to participate

All animal procedures were performed according to the national regulations and approved by the Institutional Animal Care and Use Committee (IACUC) of Shenzhen People’s Hospital, Guangdong Province, China (No. AUP-220516-SY-0242-01).

## Conflict of interest

The authors declare no competing interests.

## Supplementary Material

Supplemental_Information_tkad058
