# Supplementary material for: Exosomal miR-4645-5p from hypoxic bone marrow mesenchymal stem cells facilitates diabetic wound healing by restoring keratinocyte autophagy
Source: Burns Trauma. 2024 Jan 17;12:tkad058. doi: 10.1093/burnst/tkad058 (PMC10796268; doi:10.1093/burnst/tkad058)
Supplement: Supplemental_Information_tkad058 [file supplemental_information_tkad058.docx]

**Exosomal miR-4645-5p from hypoxic BMSCs facilitates diabetic wound healing by restoring keratinocyte autophagy**


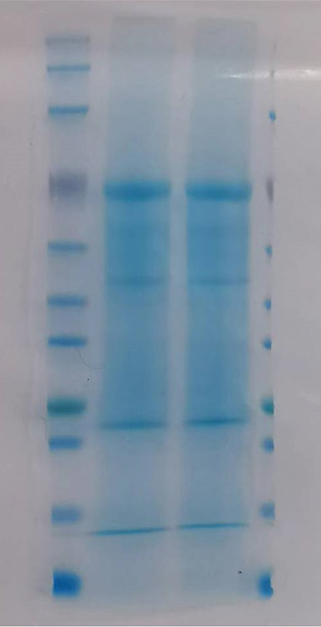


**Figure S1.** The result of coomassie blue gel staining showed that the total loaded protein amounts were equivalent in the first place.


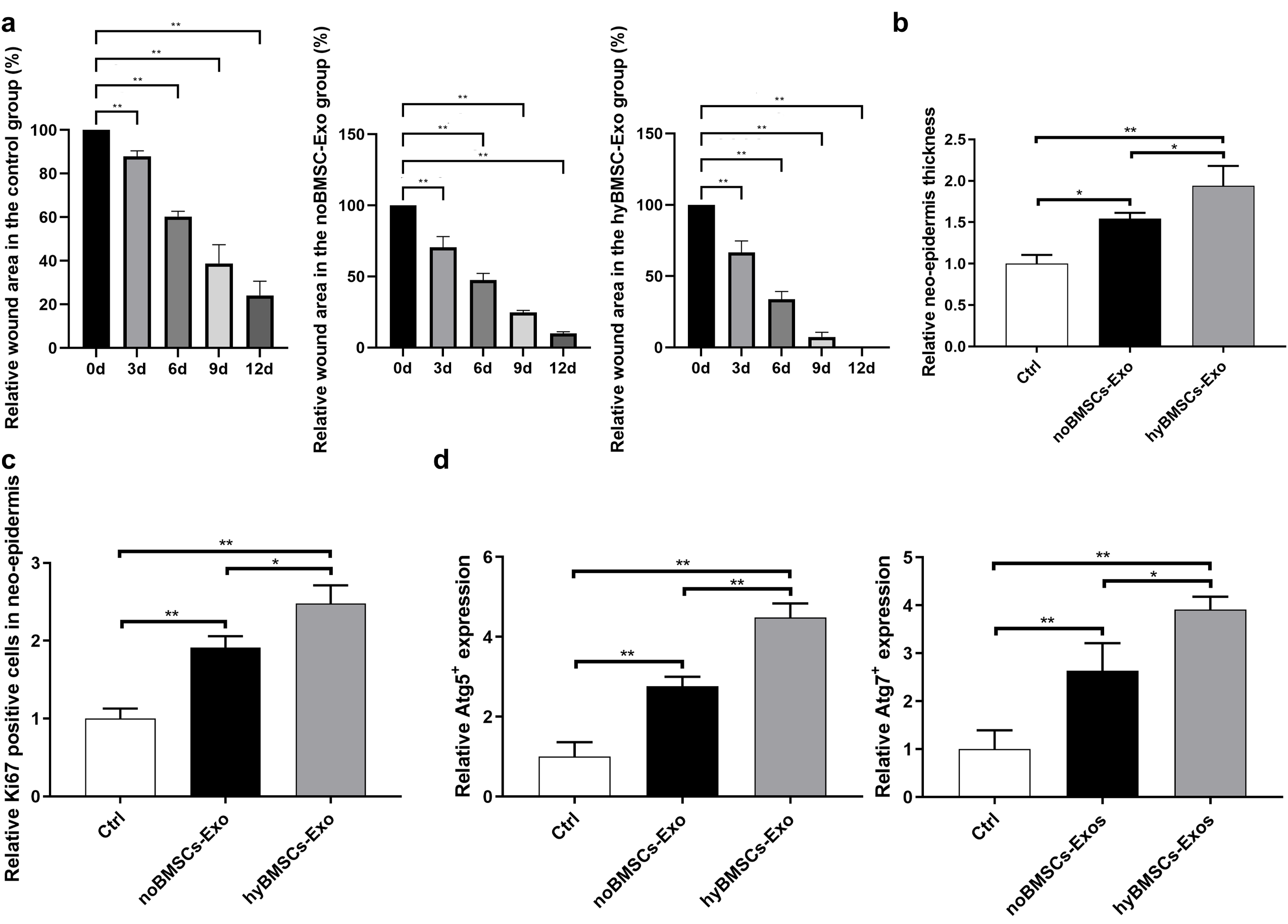


**Figure S2.** hyBMSC-Exo transplantation promotes diabetic wound healing. (**a**) Quantification of the relative wound area (%) in each group of Figure 2A (n=6). (**b**) Quantification of Figure 2C (n=6). (**c**) Quantification of Figure 2D (n=6). (**d**) Quantification of Figure 2G (n=6). The data were presented as mean ± SD. **p* < 0.05, ***p* < 0.01. noBMSC-Exo normoxic BMSC-sourced exosome, hyBMSC-Exo hypoxic BMSC-sourced exosome, Ctrl control, Atg5 autophagy-related 5, Atg7 autophagy-related 7.

**
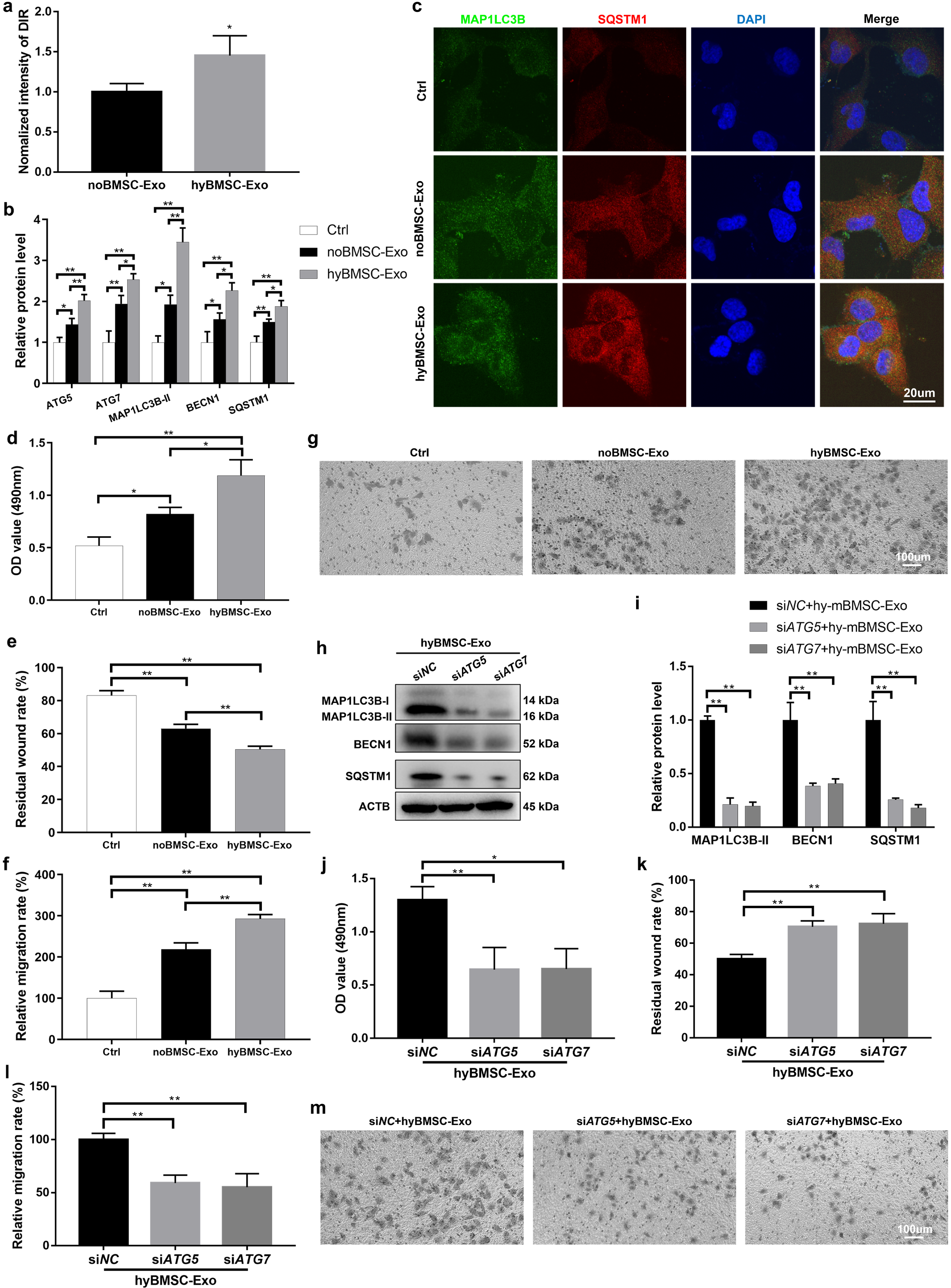
**

**Figure S3.** hyBMSC-Exo-induced autophagy contributes to the activation of HaCaT proliferation and migration. (**a**) Statistical evaluation of fluorescence intensities for DIR-labeled noBMSC-Exos or hyBMSC-Exos taken by HaCaT cells. (**b**) Quantitative analysis of western blotting analysis for autophagy-associated proteins in the HaCaT cells treated with hyBMSC-Exo or noBMSC-Exo or without exosome treatments for 24 h. (**c**) Immunofluorescence staining for MAP1LC3B (green), SQSTM1 (red), and DAPI (blue, nucleus) staining of HaCaT cells in the three groups (scale bar: 20 μm). (**d**) The above HaCaT cell proliferation was assessed using MTS assay. (**e and f**) Quantitative analysis of relative wound rate and relative migration rate in the above three groups. (**g**) Representative images of matrigel (transwell) assay with HaCaT cells in the above three groups(scale bar: 100 μm). (**h and i**) Western blotting analysis for MAP1LC3B-I/II, BECN1, and SQSTM1 in HaCaT cells transfected with si*NC*, si*ATG5*, and si*ATG7* after treatment with hyBMSC-Exos for 24 h. (**j**) HaCaT cell proliferation 24 h after hyBMSC-Exo treatment was measured using MTS assay. (**k and l**) Quantitative analysis of relative wound rate and relative migration rate for HaCaT cells after hyBMSC-Exo treatment. (**m**) Representative images of matrigel (transwell) assay with HaCaT cells after hyBMSC-Exo treatment (scale bar: 100 μm). The data were presented as mean ± SD, all data are from n = 3 independent experiments. **p* < 0.05, ***p* < 0.01. noBMSC-Exo normoxic BMSC-sourced exosome, hyBMSC-Exo hypoxic BMSC-sourced exosome, Ctrl control, ATG5 autophagy-related 5, ATG7 autophagy-related 7, ACTB β-actin, MAP1LC3B-I/II microtubule-associated protein 1 light chain 3 beta, BECN1 beclin 1, SQSTM1 sequestosome 1.

**
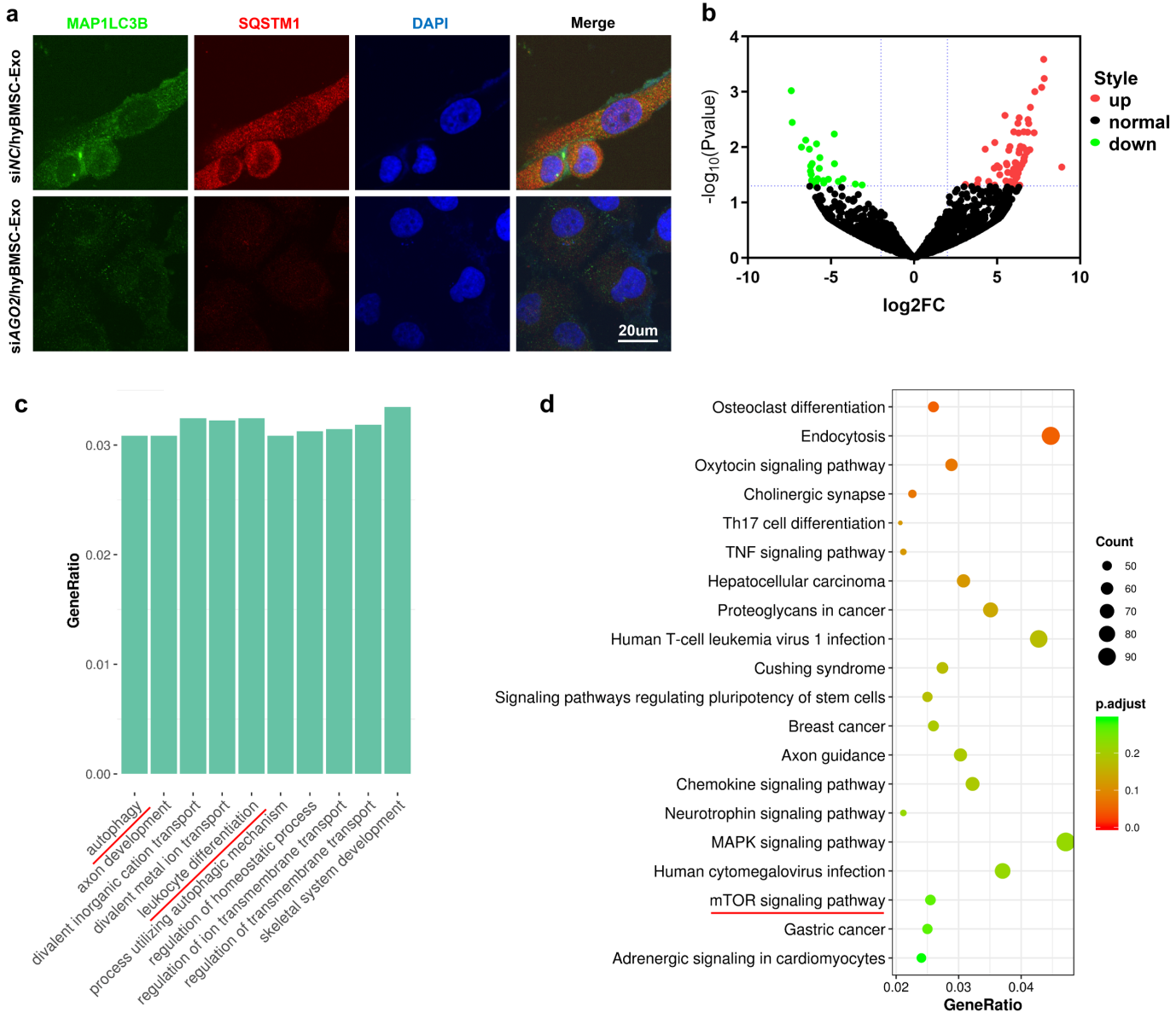
**

**Figure S4.** MiRNA sequencing of exosomes from hypoxia- and normoxia-treated BMSCs showed considerable differences in the composition of exosomal miRNAs. (**a**) Immunofluorescence staining for MAP1LC3B (green), SQSTM1 (red), and DAPI (blue, nucleus) staining of HaCaT cells in the two groups (scale bar: 20 μm). (**b**) Volcano plot of differentially expressed miRNAs depicting up- and down-regulated miRNAs. The independent samples t-test was used to determine if there are significant difference between two sample means. The *P* values were corrected by FDR method. (**c**) GO analysis of target genes of the differentially expressed miRNAs indicated that they were enriched in biological process related to “autophagy” and “process utilizing autophagic mechanism”. (**d**) KEGG pathway enrichment analysis of target genes of the differentially expressed miRNAs between the noBMSC-Exo and hyBMSC-Exo groups. hyBMSC-Exo hypoxic BMSC-sourced exosome, MAP1LC3B-I/II microtubule-associated protein 1 light chain 3 beta, SQSTM1 sequestosome 1, GO Gene Ontology, KEGG Kyoto Encyclopedia of Genes and Genomes.


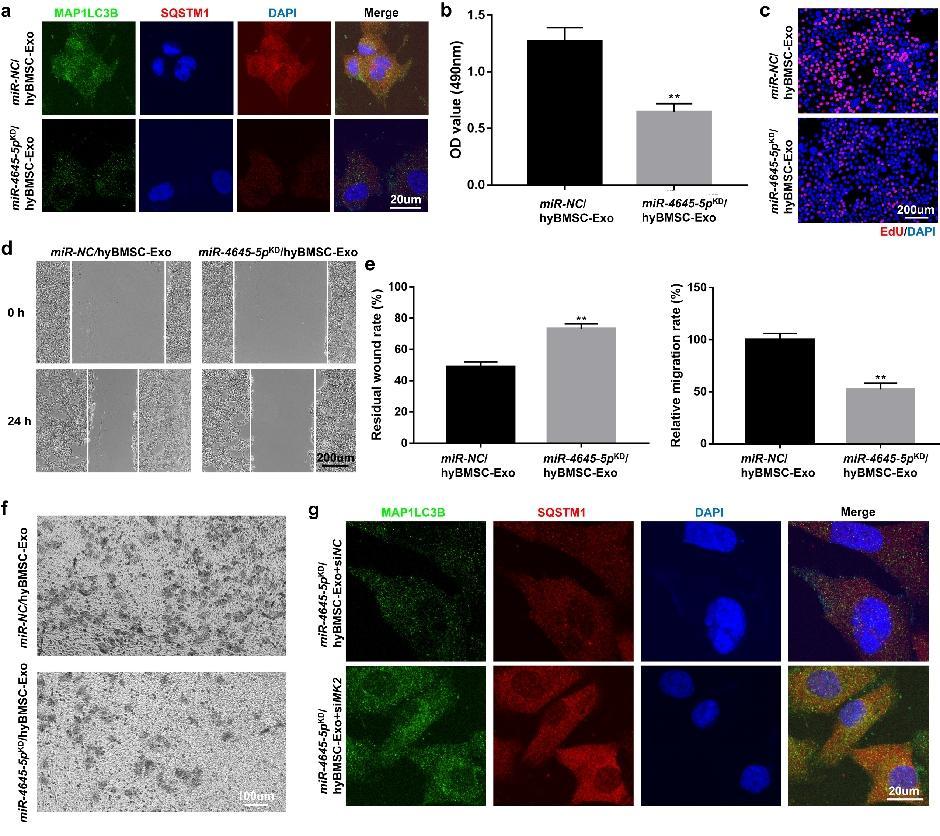


**Figure S5.** miR-4645-5p is a major contributor to the pro-autophagy effect of hyBMSC-Exos. (**a**) Immunofluorescence staining for MAP1LC3B (green), SQSTM1 (red), and DAPI (blue, nucleus) staining of HaCaT cells after miR-NC/hyBMSC-Exo or miR-4645-5pKD/hyBMSC-Exo treatment for 24 h. (scale bar: 20 μm). (**b** and **c**) HaCaT cell proliferation 24 h after miR-NC/hyBMSC-Exo or miR-4645-5p^KD^/hyBMSC-Exo treatment was measured using MTS and EdU assays (scale bar: 200 μm). (**d**-**f**) HaCaT cell migration was assessed after miR-NC/hyBMSC-Exo or miR-4645-5p^KD^/hyBMSC-Exo treatment using wound healing and matrigel (transwell) assays (scale bar: 200 μm). (**g**) Immunofluorescence staining for MAP1LC3B (green), SQSTM1 (red), and DAPI (blue, nucleus) staining of transfected HaCaT cells after miR-NC/hyBMSC-Exo or miR-4645-5pKD/hyBMSC-Exo treatment for 24 h. (scale bar: 20 μm). The data were presented as mean ± SD, all data are from n = 3 independent experiments. **p* < 0.05, ***p* < 0.01. hyBMSC-Exo hypoxic BMSC-sourced exosome, miR/miRNA microRNA, MAP1LC3B-I/II microtubule-associated protein 1 light chain 3 beta, SQSTM1 sequestosome 1, EdU 5-Ethynyl-2’-deoxyuridine

**
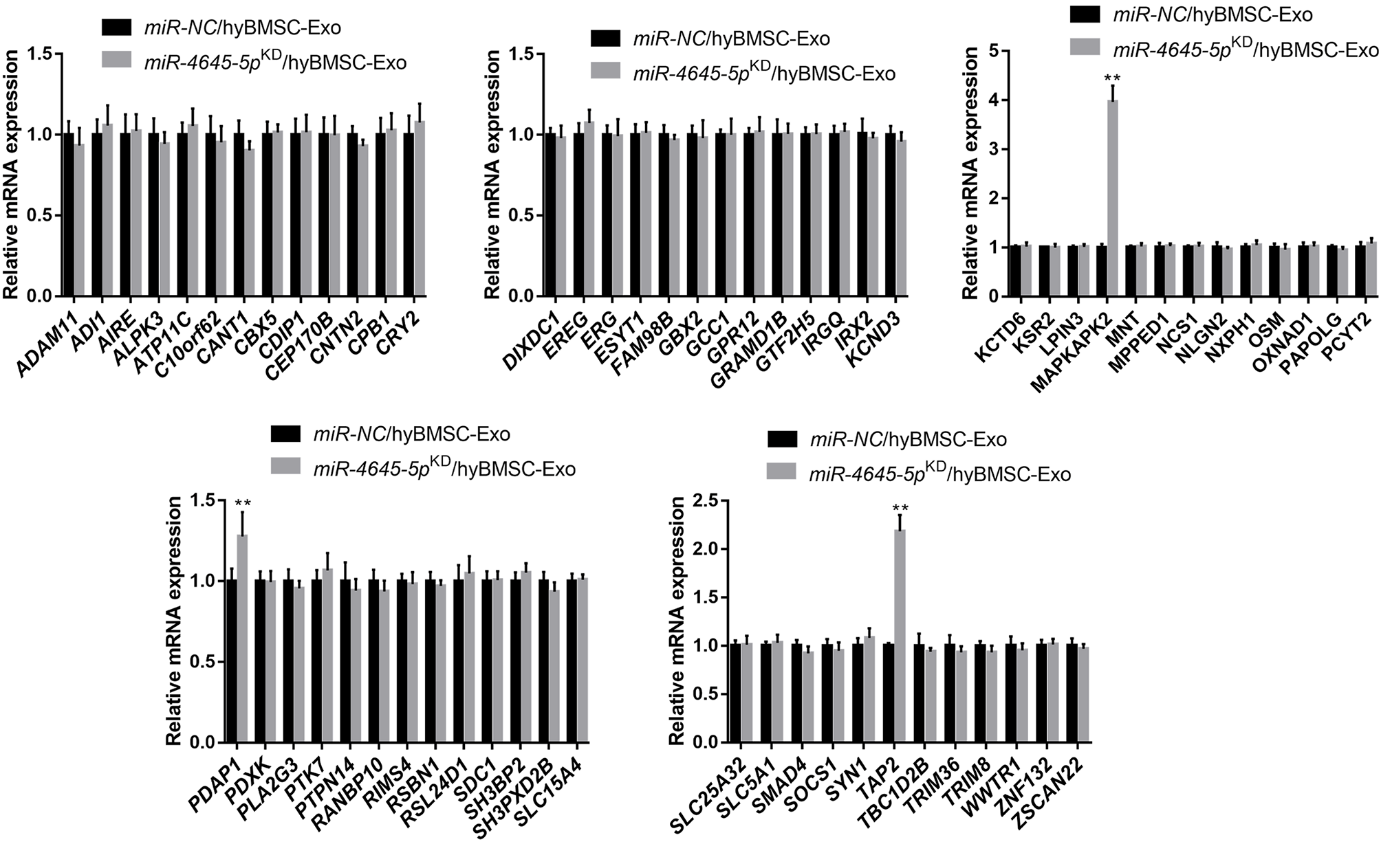
**

**Figure S6.** Comparison of the expression levels of 64 target genes of miR-4645-5p in HaCaT cells after miR-NC/hyBMSC-Exo and miR-4645-5p^KD^/hyBMSC-Exo treatments. The independent samples t-test was used to determine if there are significant difference between two sample means. The data were presented as mean ± SD, all data are from n = 3 independent experiments. **p* < 0.05, ***p* < 0.01. hyBMSC-Exo hypoxic BMSC-sourced exosome, miR/miRNA microRNA.


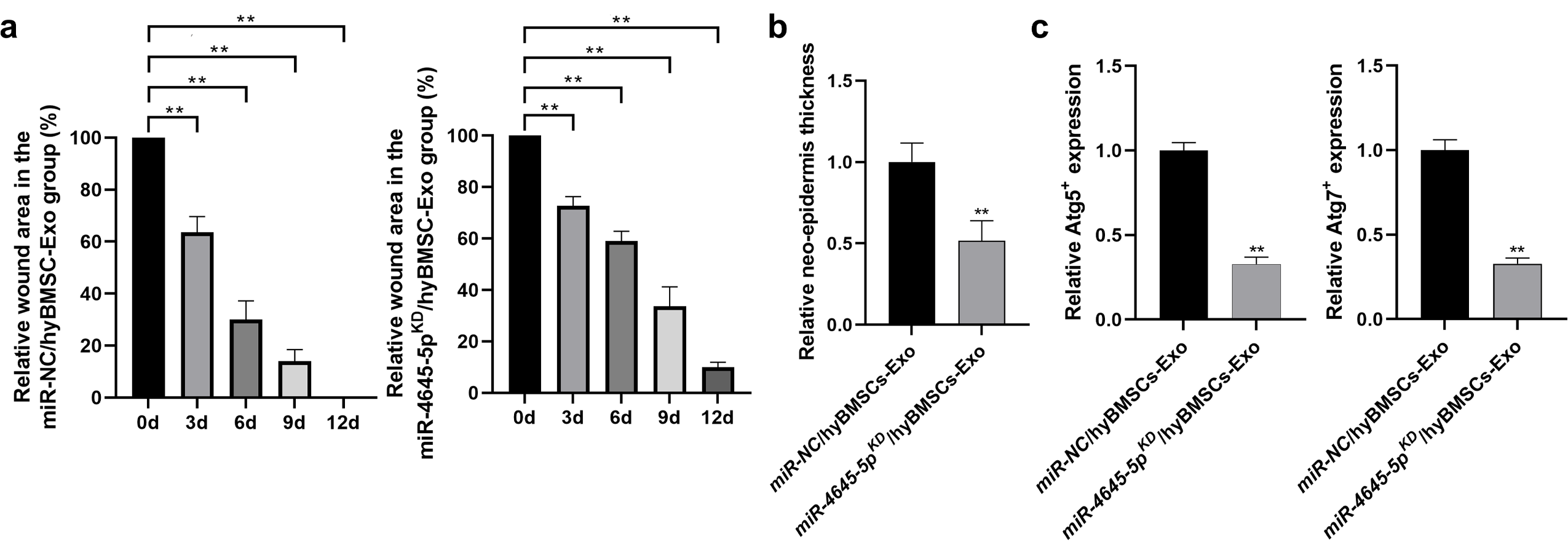


**Figure S7.** Exosomal miR-4645-5p contributes to the therapeutic effect of hyBMSC-Exos in diabetic wound healing. (**a**) Assessment of wound area in mice treated with miR-NC/hyBMSC-Exos or miR-4645-5p^KD^/hyBMSC-Exos (n = 6) at 0, 3, 6, 9, and 12 d post-wounding. (**b**) Quantification of Figure 6c (n=6). (**c**) Quantification of Figure 6D (n=6). The data were presented as mean ± SD. **p* < 0.05, ***p* < 0.01. miR/miRNA microRNA, hyBMSC-Exo hypoxic BMSC-sourced exosome, K14 Keratin 14.


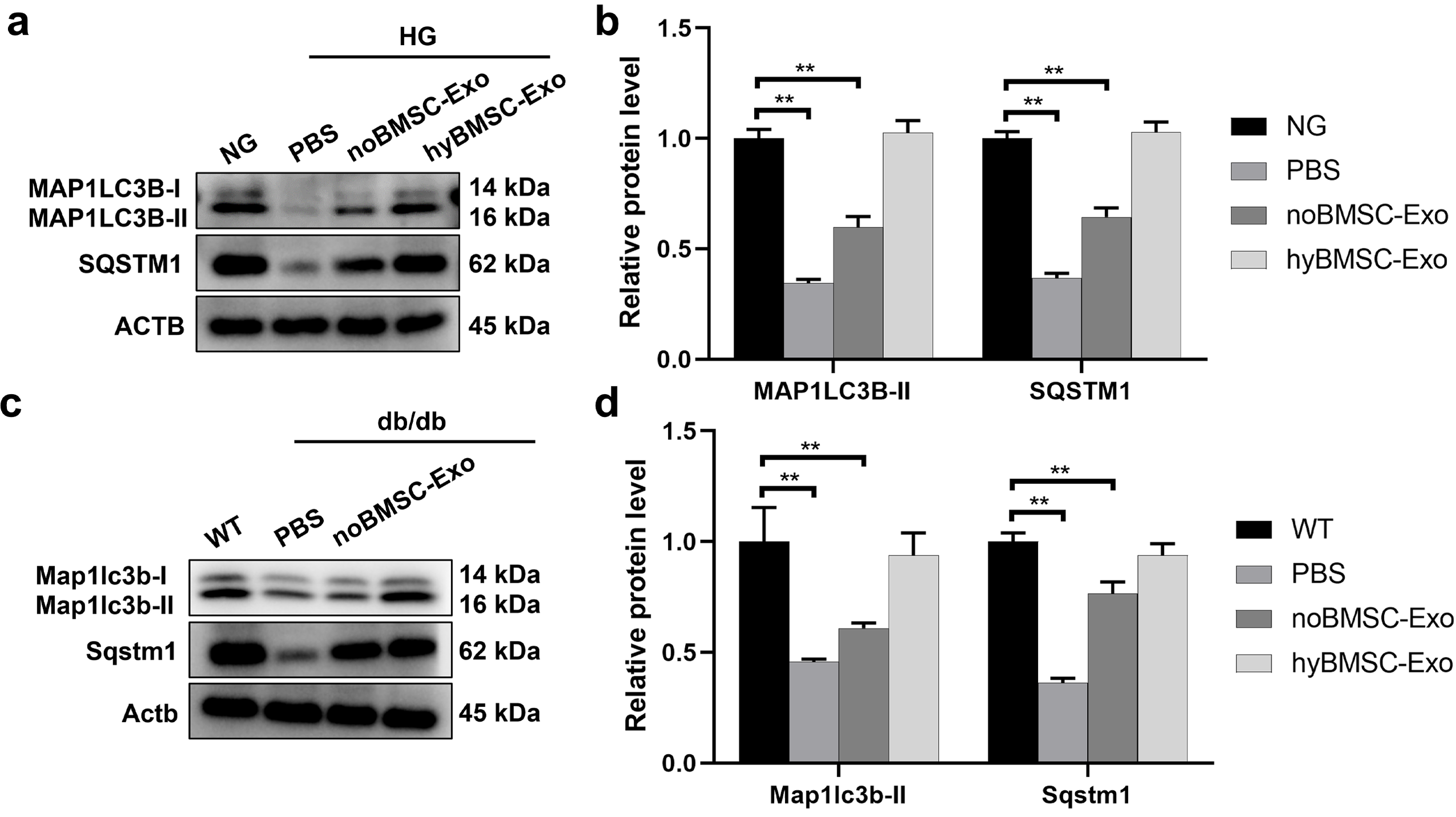
**Figure S8.** (**a** and **b**) Western blotting analysis of MAP1LC3B-I/II and SQSTM1 in HG-treated HaCaT cells with different exosomes treatment or not, NG-treated HaCaT cells as a control. (**c** and **d**) Western blotting analysis of Map1lc3b-I/II and Sqstm1 in db/db mouse wound models under different exosomes treatment or not, WT mouse wound models as a control. The data were presented as mean ± SD, all data are from n = 3 independent experiments. **p* < 0.05, ***p* < 0.01. NG normal glucose, HG high glucose, noBMSC-Exo normoxic BMSC-sourced exosome, hyBMSC-Exo hypoxic BMSC-sourced exosome, MAP1LC3B/Map1lc3b-I/II microtubule-associated protein 1 light chain 3 beta, BECN/Becn1 beclin 1, SQSTM1/Sqstm1 sequestosome 1, ACTB/Actb β-actin.


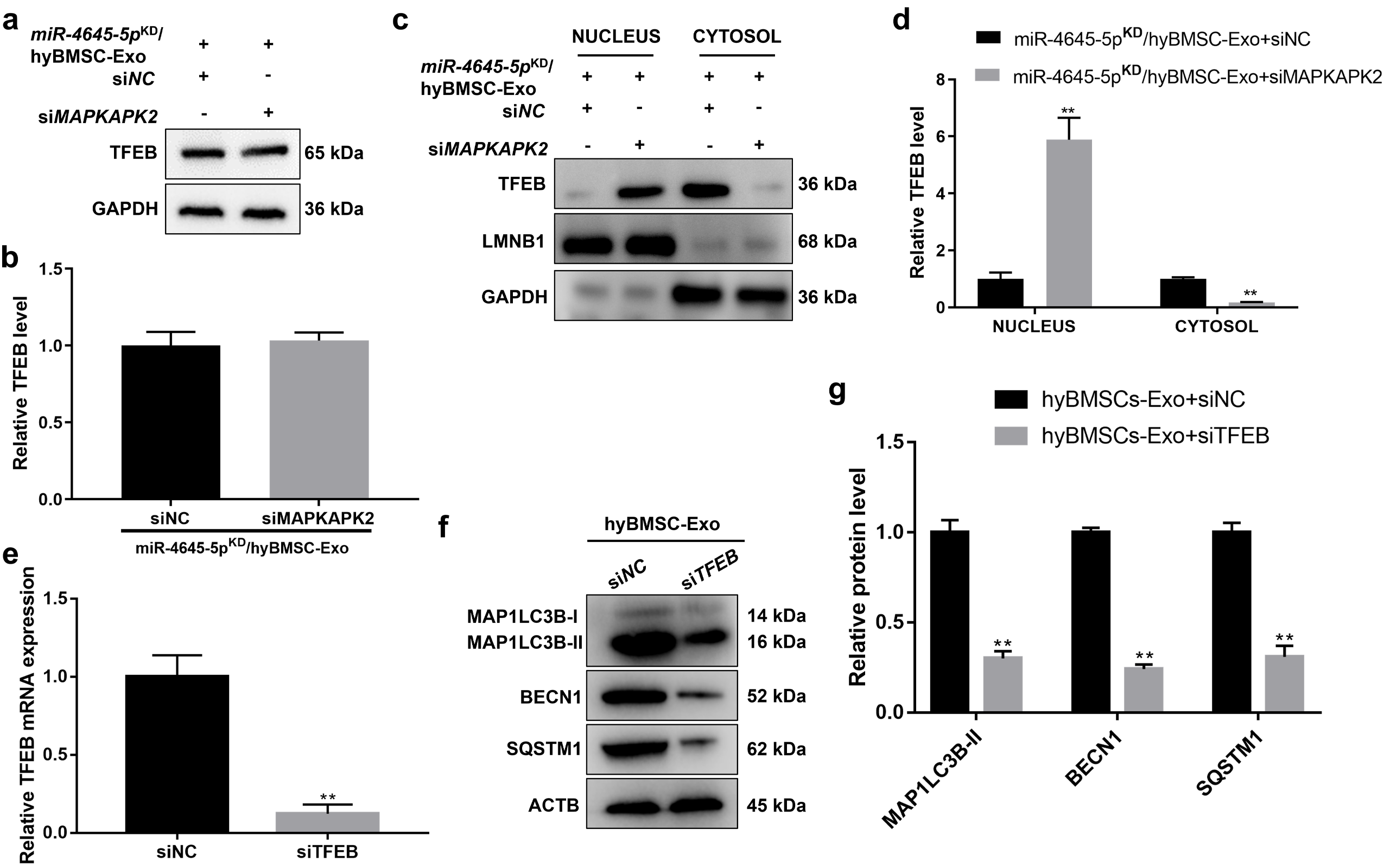


**Figure S9.** mTORC1 inactivation induced the increased expression of the autophagy-related protein by increase nuclear translocation of TFEB. (**a** and **b**) Western blotting analysis of TFEB expression levels in transfected HaCaT cells after miR-NC/hyBMSC-Exo or miR-4645-5p^KD^/hyBMSC-Exo treatments for 24 h. (**c** and **d**) Western blotting and quantitative analysis were used to examine the proportion of TFEB expression in the nucleus and cytoplasm of transfected HaCaT cells treated with miR-NC/hyBMSC-Exos or miR-4645-5p^KD^/hyBMSC-Exos for 24 h. (**e**) qRT-PCR for *TFEB* mRNA levels in HaCaT cells transfected with si*NC* or si*TFEB*. (**f** and **g**) Western blotting analysis of autophagy-associated proteins in transfected HaCaT cells after hyBMSC-Exo treatment for 24 h. The independent samples t-test was used to determine if there are significant difference between two sample means. The data were presented as mean ± SD, all data are from n = 3 independent experiments. **p* < 0.05, ***p* < 0.01. TFEB transcription factor EB, hyBMSC-Exo hypoxic BMSC-sourced exosome, miR/miRNA microRNA, MAPKAPK2 mitogen-activated protein kinase-activated protein kinase 2, MAP1LC3B-I/II microtubule-associated protein 1 light chain 3 beta, BECN beclin 1, SQSTM1 sequestosome 1, LMNB1 lamin B1.

| **Gene** | **Forward** | **Reverse** |
| --- | --- | --- |
| ATG5 | 5’-GCTTCGAGATGTGTGGTTTGG-3’ | 5’-ACTTTGTCAGTTACCAACGTCA-3’ |
| ATG7 | 5'- TTTGCTATCCTGCCCTCT-3' | 5'- TGCCTCCTT TCTGGTTCT-3' |
| Ago2 | 5’- GTTTGACGGCAGGAAGAATCT-3' | 5’- AGGACACCCACTTGATGGACA-3' |
| GAPDH | 5’-GTATCGTGGAAGGACTCATGAC-3’ | 5’-ACCACCTTCTTGATGTCATCAT-3’ |
| ADAM11 | 5’-AACCCAGCCGTCTGGTTAG-3’ | 5’-TCGTACACATCCACGATCCGT-3’ |
| ADI1 | 5’-GGAGCATTTGCACTTGGACG-3’ | 5’-CGTAGCTCCTTTCAGCAAGAGA-3’ |
| AIRE | 5’-AACGGGATTCAGACCATGTCA-3’ | 5’-GGTCTGAGGCATTGGTTCGG-3’ |
| ALPK3 | 5’-CAGCTCAGACGAGCCTGAC-3’ | 5’-GGGACAGTGCTATTGTATCCAC-3’ |
| ATP11C | 5’-AGTACGTGATACCATTGCACTG -3’ | 5’-GTGGCATCGTCTTTTGCTTTC-3’ |
| C10orf62 | 5’-AAGTCACCAGAATCCCATAAAGC-3’ | 5’-GGGAAAAAGCATTTTGGTCCATT-3’ |
| CANT1 | 5’-GGAAGGTGATCCTGACGTTCT-3’ | 5’-AGTCCTCGATGTTCAGCCCA-3’ |
| CBX5 | 5’-CTAGACAGGCGCGTGGTTAAG-3’ | 5’-GCCTTGTCGAAGTTACCGC-3’ |
| CDIP1 | 5’-ATTGGCTTGATGAATTTCGTGC-3’ | 5’-GTGGGGCTTAACACATTATACCA-3’ |
| CEP170B | 5’-TGTTATCTAGGAAACCGCTTGC-3’ | 5’-GCAGCGTTTGTGGATGATGA-3’ |
| CNTN2 | 5’-CACTACCCAGGCTTGTCCTAC-3’ | 5’-CGCTGGCATTGTTGTCGAG-3’ |
| CPB1 | 5’-GCAGTGTTATCGGAACCACAT-3’ | 5’-CTGTAGCGCCGGTAAATGG-3’ |
| CRY2 | 5’-AACCACGACGAGACCTACG-3’ | 5’-GCAGCCCTCTTAGCTTTAGCA-3’ |
| DIXDC1 | 5’-CTTACAGGGGATAAAGGATGCCT-3’ | 5’-CCACCAAGGAATCCTAGCATGT-3’ |
| EREG | 5’-GTGATTCCATCATGTATCCCAGG-3’ | 5’-GTGTCATTGTACCAGTTGGCG-3’ |
| ERG | 5’-CGTGCCAGCAGATCCTACG-3’ | 5’-GGTGAGCCTCTGGAAGTCG-3’ |
| ESYT1 | 5’-CTGGCGGTGCTGACTTCATT-3’ | 5’-CTCGAAGGCTCCGTTCTTTCT-3’ |
| FAM98B | 5’-TGGAGGCGCTGGGGTATAAA-3’ | 5’-GTGCGTCACATCCTTGAAGTC-3’ |
| GBX2 | 5’-CTCACCTCTACGCTCATGGC-3’ | 5’-GAGTTGGCGTTCATTCGGG-3’ |
| GCC1 | 5’-CACGGCTCAAGGATGTGGTC-3’ | 5’-GGAAGACCACAAAAGCAAGGC-3’ |
| GPR12 | 5’-GGGCTGCCTCGGGATTATTTA-3’ | 5’-CAAACTGCGGTAGTCTCCGTT-3’ |
| GRAMD1B | 5’-AGAAGGGCTCAGATCACTCCT-3’ | 5’-TGAAGCCCCAGCCGTAGAA-3’ |
| GTF2H5 | 5’-AAGACATTGATGACACTCACGTC-3’ | 5’-GTCACCCAAGACTGGATCACA-3’ |
| IRGQ | 5’-TGCGACAAGGATGTGGAGAC-3’ | 5’-GTGGGCGATGTTTTCACTGAG-3’ |
| IRX2 | 5’-AAGGACGAGAGTCCCGACAA-3’ | 5’-CACAAGACAATGTCCCAGGGG-3’ |
| KCND3 | 5’-GGAGACATGGTGCCTAAGACG-3’ | 5’-GAGTTGACGATTGGGAGCCT-3’ |
| KCTD6 | 5’-ACGCAACTAACCATCACCACT-3’ | 5’-GCCATTCATGTCAGAGCTACACT-3’ |
| KSR2 | 5’-GTGGAGAAATACGGAGCCAAC-3’ | 5’-AGGTGATACAACTCGTTCGTAGT-3’ |
| LPIN3 | 5’-GGGGCCAGAAGATGGAGTG-3’ | 5’-CATTGTCCAAGTCGTAGAGCTT-3’ |
| MK2 | 5’-CCAGGAGAAATTCGCCCTCAA-3’ | 5’-CTCGTGGGATACCGACAGC-3’ |
| MNT | 5’-TGGACGTACTGGAGATTGACC-3’ | 5’-TCTGGCAGGACTACAGCCTC-3’ |
| MPPED1 | 5’-CCGGAGAACTATGAGAATGTGC-3’ | 5’-AGGTGGACCCTAAGAGAAACTTC-3’ |
| NCS1 | 5’-CCCACCAAGTTTGCCACATTT-3’ | 5’-GAGCACGAGTGATCCGTGA-3’ |
| NLGN2 | 5’-TCAACTACCGTCTTGGGGTG-3’ | 5’-CCTTGAGCCGAGAGTCCTATT-3’ |
| NXPH1 | 5’-TGCTGGTACGTGCTTTTCCTC-3’ | AGCGCATTGGGCATACTCAT-3’ |
| OSM | 5’-CACAGACTGGCCGACTTAGAG-3’ | 5’-GCTCAGGGCAATCCAAGTTTT-3’ |
| OXNAD1 | 5’-CACTTTGCGCCACCTTACTCT-3’ | 5’-GTGACAGGAGTCGGCTGATAG-3’ |
| PAPOLG | 5’-TCGCAGCTTAAATGGTTGTAGAG-3’ | 5’-CCAGCCACATAGATGACTGTCT-3’ |
| PCYT2 | 5’-ACCGGGGTATCCCAGTTCC-3’ | 5’-CGAACTTGCTGGGAGTGTAGAA-3’ |
| PDAP1 | 5’-CAAAGGAGCTTTCGAGGAGAG-3’ | 5’-GAGAGCCTAACCAAATACAGAGC-3’ |
| PDXK | 5’-CTCACAGGTTATACGAGGGACA-3’ | 5’-GTGAAGCGGTGATAGATCCCC-3’ |
| PLA2G3 | 5’-TGTGGAGTTGGAGATTCTGCT-3’ | 5’-CCATATTTGCCCTCAGTAGCTC-3’ |
| PTK7 | 5’-ACACTTCGTTGCCACATTGAT-3’ | 5’-TGGTAAATCCGGCTAAAGTTGG-3’ |
| PTPN14 | 5’-ATGCCTTTTGGTCTGAAGCTC-3’ | 5’-CCCTGTGCTTTCCACCGAC-3’ |
| RANBP10 | 5’-AACCTCCGCGTCCACTACA-3’ | 5’-GGTACGAACAGCCTCTCTTACA-3’ |
| RIMS4 | 5’-GGATGTGGAGATCGGTCTGC-3’ | 5’-TGGAAGGATCTGCATTAGGGA-3’ |
| RSBN1 | 5’-CGATCTCAAGCACAAGGACAA-3’ | 5’-ATCGGCTCTCTGGGTTTTACC-3’ |
| RSL24D1 | 5’-GACACGGCATGATGTTCGTC-3’ | 5’-GAGGCAGTCGAAGCTCTCG-3’ |
| SDC1 | 5’-ACGGCTATTCCCACGTCTC-3’ | 5’-TCCTCGTCTATGTTGTCCTCAC-3’ |
| SH3BP2 | 5’-CATTGGCCTGTCCCTATGAAG-3’ | 5’-GCGAGCGACTTTGGTCTTCT-3’ |
| SH3PXD2B | 5’-TGGAGGTGAAGGTGCTAGAC-3’ | 5’-TGGGATGACGAAACTCACCTG-3’ |
| SLC15A4 | 5’-TGGGAGCGATCCTGTCGTTA-3’ | 5’-GGTCAATGAATTGCGCTTCCA-3’ |
| SLC25A32 | 5’-TCCCCACACCGACAATATAAAGG-3’ | 5’-GGCTTCCTGGTACTATCTGTGC-3’ |
| SLC5A1 | 5’-TACCTGAGGAAGCGGTTTGGA-3’ | 5’-GCGAGGATTGCGCTTCTTTTTA-3’ |
| SMAD4 | 5’-CTCATGTGATCTATGCCCGTC-3’ | 5’-CCATGCGATGTTCCAAACAGC-3’ |
| SOCS1 | 5’-TTTTCGCCCTTAGCGTGAAGA-3’ | 5’-CGTAGCTGGATGACCATTCTC-3’ |
| SYN1 | 5’-AGTTCTTCGGAATGGGGTGAA-3’ | 5’-CAGCAGGAATACAGCCCAC-3’ |
| TAP2 | 5’-TGGACGCGGCTTTACTGTG-3’ | 5’-CGGTAGTTTCGGATGCCATAGTT-3’ |
| TBC1D2B | 5’-CCGCTGCTACCTTTACTATTTCA-3’ | 5’-TCAGCTCGCCCAGAAAAAGG-3’ |
| TRIM36 | 5’-TTCAACGATGTGGGATCAGACA-3’ | 5’-AATCTCCCGTCGAAGGACACT-3’ |
| TRIM8 | 5’-CGTGGAGATCCGAAGGAATGA-3’ | 5’-CGAGAAGATGTCTGCCGAGA-3’ |
| WWTR1 | 5’-TCCCAGCCAAATCTCGTGATG-3’ | 5’-TCCTTCCCCGTCTCTGTCG-3’ |
| ZNF132 | 5’-ATGCTGGAAAACCTTGAGCTT-3’ | 5’-TCCCCTGATAGTCCAGACCTC-3’ |
| miR-6877-3p | 5’-GACAGCCTCTGCCCTTG-3’ |  |
| miR-6824-3p | 5'-GTCTCTGGTCTTGCCACC-3' |  |
| miR-4645-5p | 5’-AGCGCCTACCAGGCAAGAAA-3’ |  |
| miR-6801-5p | 5’-CTGGTCAGAGGCAGCAG-3’ |  |
| miR-6828-3p | 5’-GATCTGCTCTCTTGTTCCCAG-3’ |  |
| miR-6759-3p | 5’-GTGACCTTTGCCTCTCCC-3’ |  |
| miR-4529-3p | 5’-ATTGGACTGCTGATGGCC-3’ |  |
| miR-1972 | 5’-GATCAGGCCAGGCACAG-3’ |  |
| miR-5706 | 5’-CGTTCTGGATAACATGCTGAAGC-3’ |  |
| miR-6822-5p | 5’-GCAGGGAACCAGTTGGG-3’ |  |
| miR-6876-3p | 5’-AGCTGTCTGTGTTTTCCTTCTCAG-3’ |  |

**Supplementary** **Table 1.** The sequences of primers used for qRT-PCR.
